# Author Correction: Identification of optimal dosing schedules of dacomitinib and osimertinib for a phase I/II trial in advanced *EGFR*-mutant non-small cell lung cancer

**DOI:** 10.1038/s41467-022-33355-0

**Published:** 2022-09-23

**Authors:** Kamrine E. Poels, Adam J. Schoenfeld, Alex Makhnin, Yosef Tobi, Yuli Wang, Heidie Frisco-Cabanos, Shaon Chakrabarti, Manli Shi, Chelsi Napoli, Thomas O. McDonald, Weiwei Tan, Aaron Hata, Scott L. Weinrich, Helena A. Yu, Franziska Michor

**Affiliations:** 1 Department of Biostatistics, Harvard T.H. Chan School of Public Health, Boston, MA USA; 2 Department of Data Science, Dana Farber Cancer Institute, Boston, MA USA; 3 Division of Solid Tumor Oncology, Department of Medicine, Thoracic Oncology Service, Memorial Sloan-Kettering Cancer Center, Weill Cornell Medical College, New York, NY USA; 4 Oncology Research and Development, Pfizer Inc, La Jolla, CA USA; 5 Massachusetts General Hospital Cancer Center, Boston, MA USA; 6 Department of Stem Cell and Regenerative Biology, Harvard University, Cambridge, MA USA; 7 The Center for Cancer Evolution, Dana-Farber Cancer Institute, Boston, MA USA; 8 Clinical Pharmacology Oncology, Global Product Development, Pfizer Inc, San Diego, CA USA; 9 The Ludwig Center at Harvard, Boston, MA USA; 10 Department of Medicine, Harvard Medical School, Boston, MA USA; 11 The Broad Institute of MIT and Harvard, Cambridge, MA USA

**Keywords:** Cancer therapy, Applied mathematics

Correction to: *Nature Communications* 10.1038/s41467-021-23912-4, published online 17 June 2021

The original version of this Article contained an error in Fig. 1b, in which the mathematics were replaced by random symbols. In addition, the original version of this Article contained an error in Fig. 4, in which the shading representing predictions and interquartile ranges from mathematical modelling predictions was missing. This has been corrected in both the PDF and HTML versions of the Article.

