# Supplementary material for: Identification of optimal dosing schedules of dacomitinib and osimertinib for a phase I/II trial in advanced EGFR-mutant non-small cell lung cancer
Source: Nat Commun. 2021 Jun 17;12:3697. doi: 10.1038/s41467-021-23912-4 (PMC8211846; doi:10.1038/s41467-021-23912-4)
Supplement: Supplementary file 1 — Supplementary Information [file 41467_2021_23912_MOESM1_ESM.pdf]

Supplementary Material

Fig S1. Schematic of the experimental plan. For each cell type, we used two distinct plate maps (each experiment was performed in triplicate) where dacomitinib and osimertinib were switched. Between-time observations were from separate plates due to cell perturbation from the luminescent signal and hence were modeled as independent observations in our statistical analysis. The EnVision plate reader was used as the instrument for the CTG read.

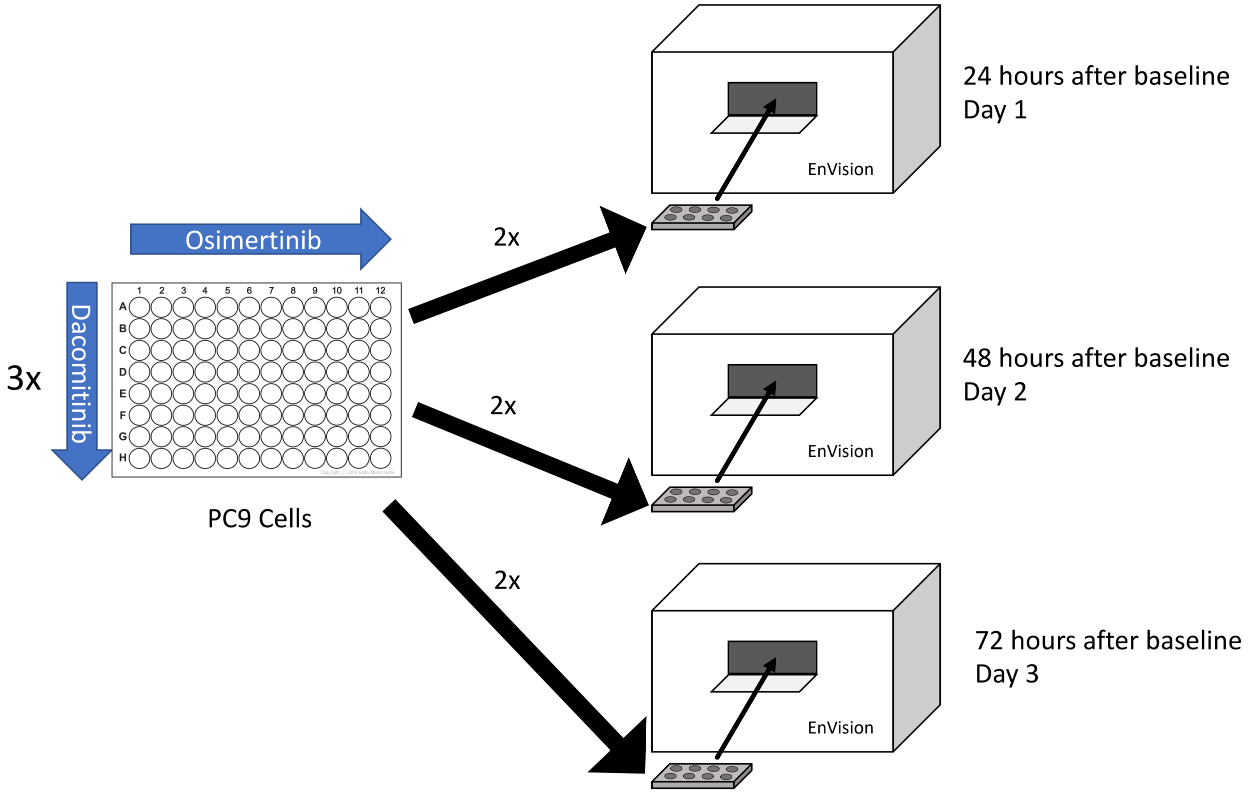

Fig S2. Fitted growth rates of CTG experiments. Experiments were done in triplicates for (a) parental cells and (b) NRAS cells. (c) Combined dacomitinib plus osimertinib does not suppress cells harboring compound T790M and C797S mutations in the used cell line. MGH 121 Res#1 cells with acquired *in cis* T790M/C797S resistance mutations[38] were treated with increasing concentrations of dacomitinib or osimertinib alone or in the presence of a fixed concentration (1mM) of osimertinib or dacomitinib, respectively. After 72 hours, cell proliferation was assessed by the CellTiter-Glo assay. Data are combined from three independent biological replicates (mean, S.E.M.).

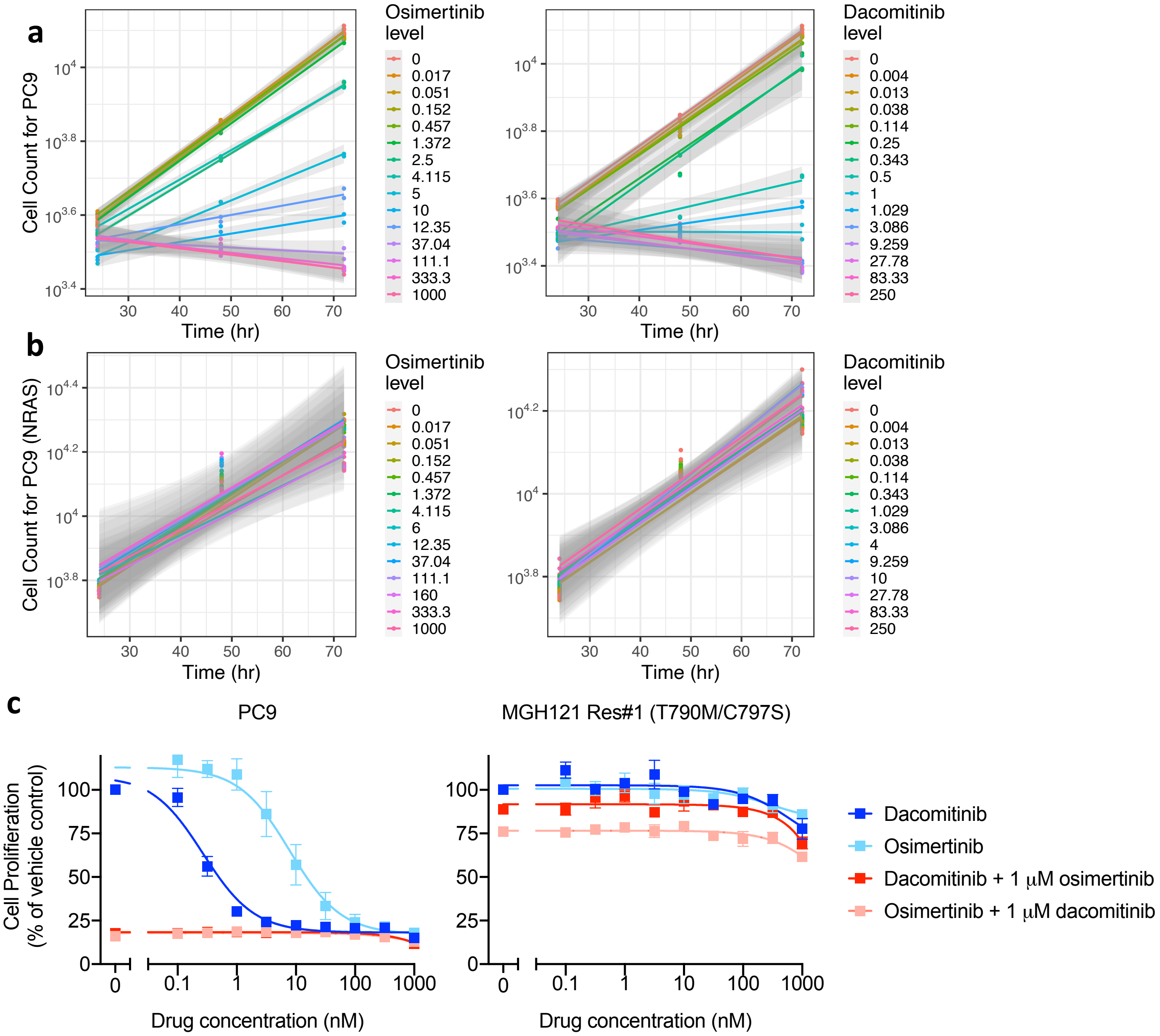

Fig S3. Inhibitory concentrations of dacomitinib and osimertinib in PC9 cells. (a) Dose-response curves of relative cell count to control over drug concentration. IC50 in PC9 cells of dacomitinib and osimertinib are 0.63 nM and 7.51 nM, respectively. The IC50 of osimertinib in the PC9-*T790M* cell line is 51.2 nM, and IC50 of dacomitinib in PC9-*C797S* cells is 11.7 nM. (b) As the concentration of dacomitinib increases, the concentration of osimertinib needed to reach IC50 decreases linearly, suggesting no interactions between these drugs. A two-sided t-test was performed when assessing deviation from additivity, which returned a p-value of 0.8517. (c-d) Signaling pathway inhibition compared with cell viability effects in response to different classes of EGFR inhibitors. (c) Immunoblot analysis of EGFR signaling in PC9 and RPC9-CL6(Del/T790M) cell lines. Cells were treated with the indicated concentrations of dacomitinib (Daco) or osimertinib (Osi) for 24h, cell lysates were prepared and analyzed by immunoblotting with antibodies to the indicated proteins and phospho-proteins. (d) Cell viability assay IC50 values for dacomitinib and osimertinib in PC9 and RPC9-CL6 cell lines after 72h of drug treatment.

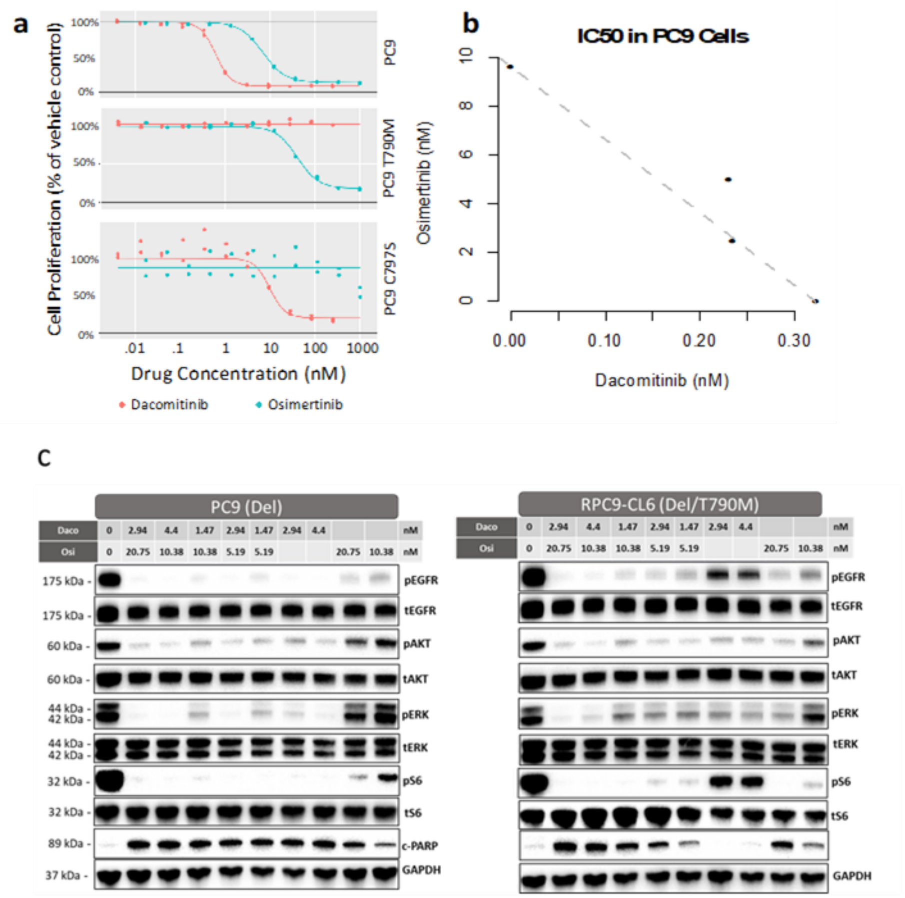

d

	PC9 (Del)	RPC9-CL9 (Del/T790M)
Dacomitinib	1 nM (n=4)	67 nM (n=3)
Osimertinib	7 nM (n=4)	10 nM (n=3)

Fig S4. Assumed death rates in simulations. (a) Death rates used in the main manuscript that were educated guesses from experimental data (negative growth rate of maximum concentration) that are constant across drug concentrations. To examine robustness, we performed simulations with death rates dependent on drug concentrations (b) modeled as a linear function of the drug concentration (i.e. $d_i = d_0 + s \times nM$, where nM is the concentration of the drug targeting the cell type of interest, and s was chosen such that maximum death rate did not surpass 0.01). We observed that our conclusions, shown in (c), from the death rates shown in (b) remained the same compared to our results using death rates shown in (a) (see **Fig 3F**).

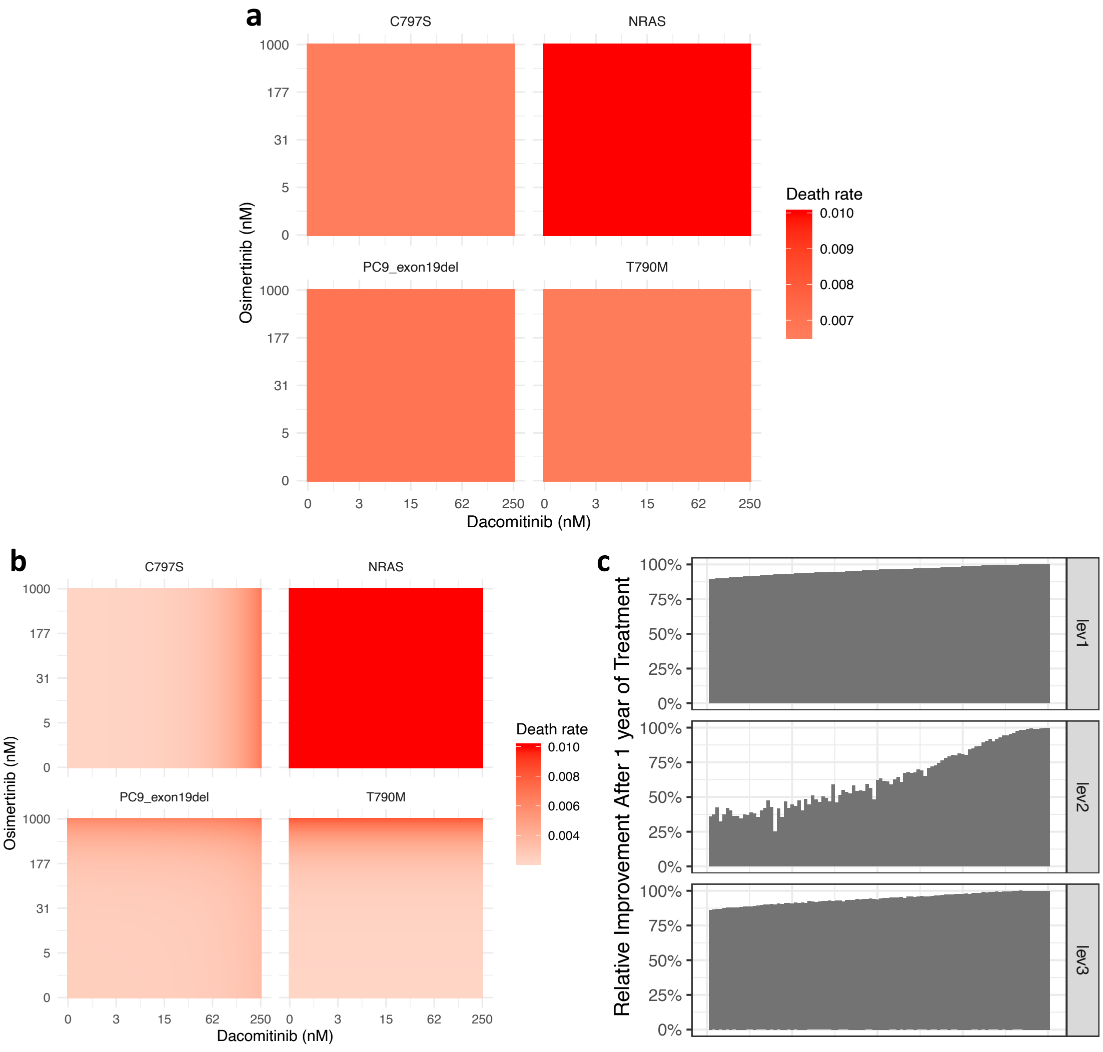

Fig S5. Fit of growth landscape in parent PC9 cell line. (a) Points represent estimated growths from CTG experiments. Red line represents the prediction from the landscape model built using design matrix shown in (b). Despite drugs not having a synergistic effect, we decided to add an interaction term to improve the fit of the growth landscape. (c) Transformations of drug concentrations that yielded best fit in growth landscape of PC9 cell line.

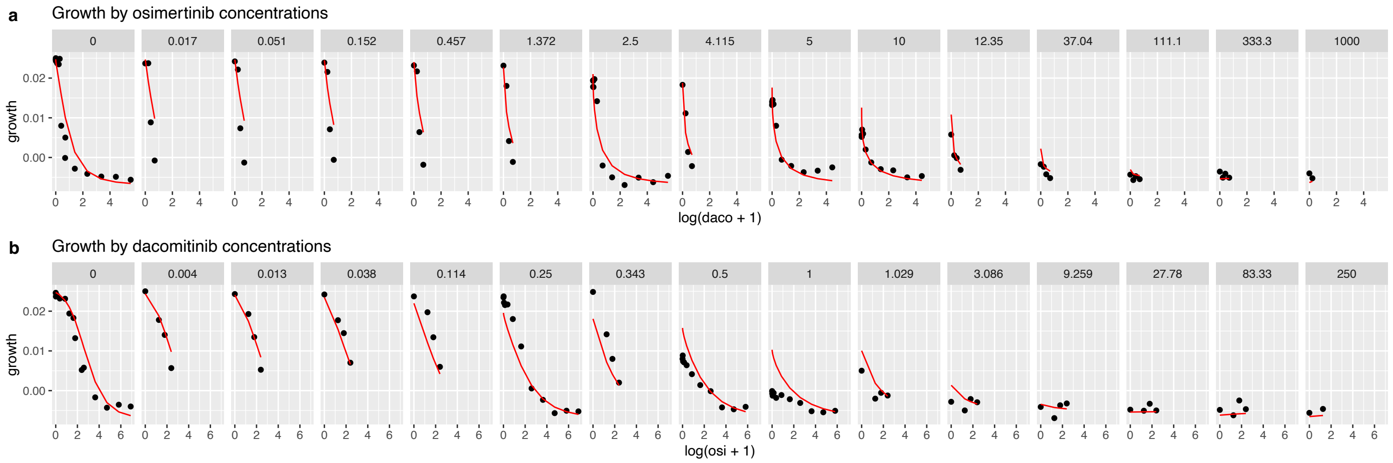

c

$$C_i = \left(\log(Daco_i + 1)^{1/3} \quad \log(Osi_i + 1)^{1/1.5} \quad \log(Daco_i + 1)^{1/3} \times \log(Osi_i + 1)^{1/1.5} \right)$$

Fig S6. Fit of growth landscape in PC9-DRH (T790M) cell line. (a) Points represent estimated growths from CTG experiments. Red line represents the prediction from the landscape model built using design matrix shown in (b). (c) Transformations of drug concentrations that yielded best fit in growth landscape of PC9-T790M+ cell line.

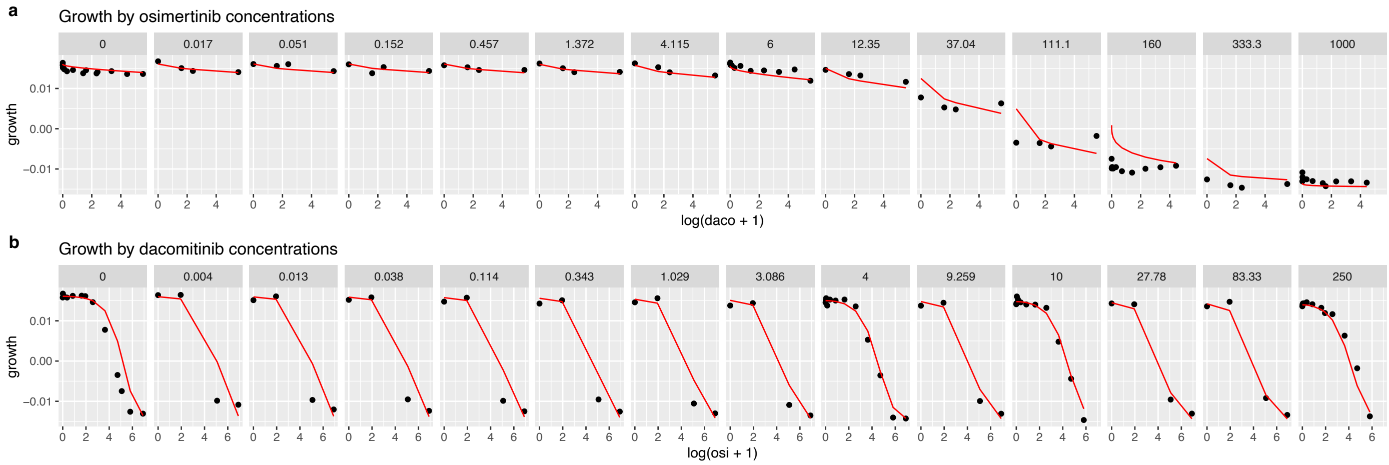

c

$$C_i = \left(\log(Daco_i + 1)^{1/3} \quad \log(Osi_i + 1)^2\right)$$

Fig S7. Fit of growth landscape in PC9 cells with C797S mutation. (a) Points represent estimated growths from CTG experiments. Red line represents the prediction from the landscape model built using design matrix shown in (b). (c) Transformations of drug concentrations that yielded best fit in growth landscape of PC9 C797S+ cell line.

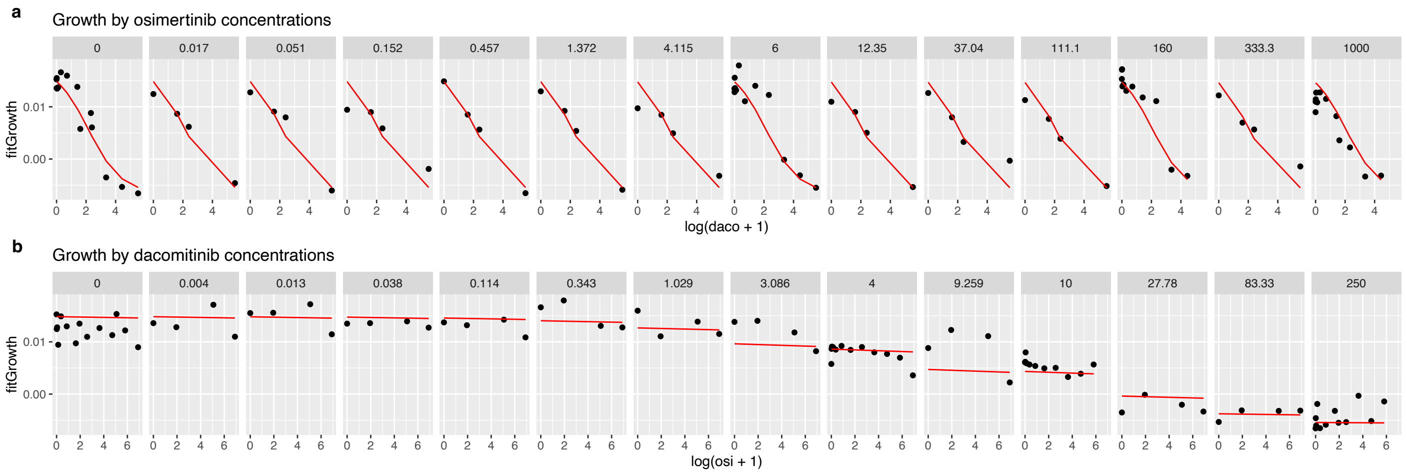

c

$$C_i = (\log(Daco_i + 1) \quad \log(Osi_i + 1))$$

Fig S8. Fit of growth landscape in PC9 cells with NRAS mutation. (a) Points represent estimated growths from CTG experiments. Red line represents the prediction from the landscape model built using design matrix shown in (b). (c) Transformations of drug concentrations that yielded best fit in growth landscape of PC9 NRAS+ cell line. (d) Birth rates of NRAS cells during combination therapy. Points represent the estimated growth rates from Fig 1C minus death rates and the contour is the predicted birth rate as a function (b) of dacomitinib and osimertinib concentration.

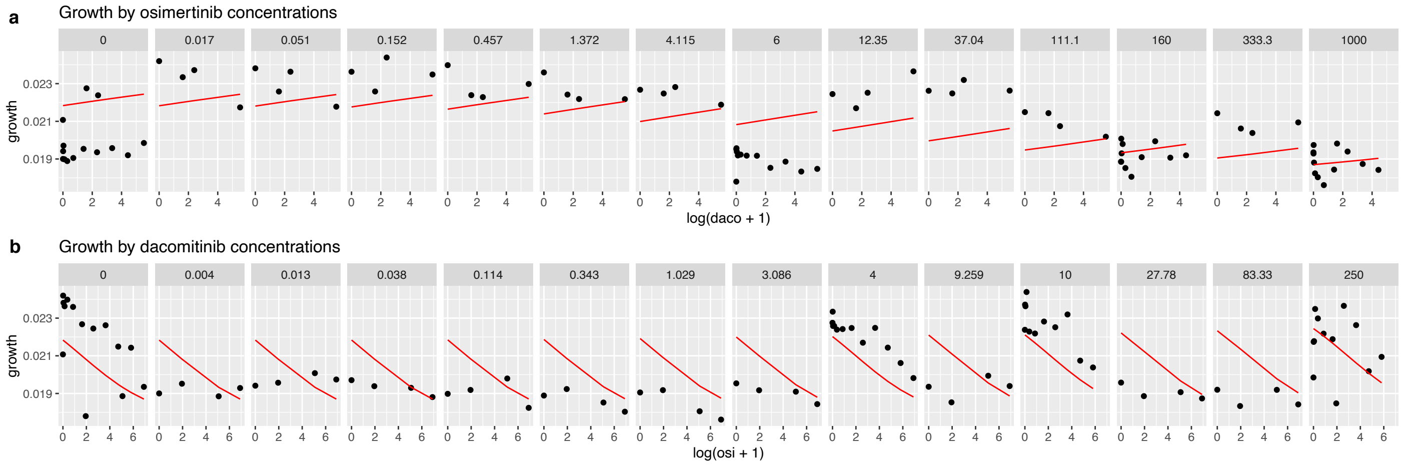

c

$$C_i = (\log(Daco_i + 1) \quad \log(Osi_i + 1))$$

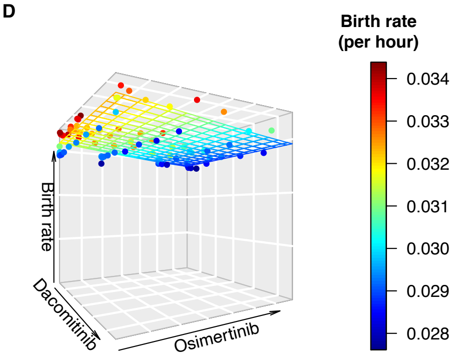

Fig S9. Initial settings in simulated patients (a) Distribution of mutation rates used in simulations. For each patient, we sampled a mutations rate for each resistance mechanism from the distributions shown. These sampled mutation rates were assumed to be constant over time. (b) Frequency of pre-existent resistance in patient simulations. T790M, a commonly observed resistance, is present in almost 80% of patients at varying frequencies, whereas NRAS is present in only 1% of patients but at a much lower frequency. Additionally, we assumed that there is no C797S pre-existence in our patient population.

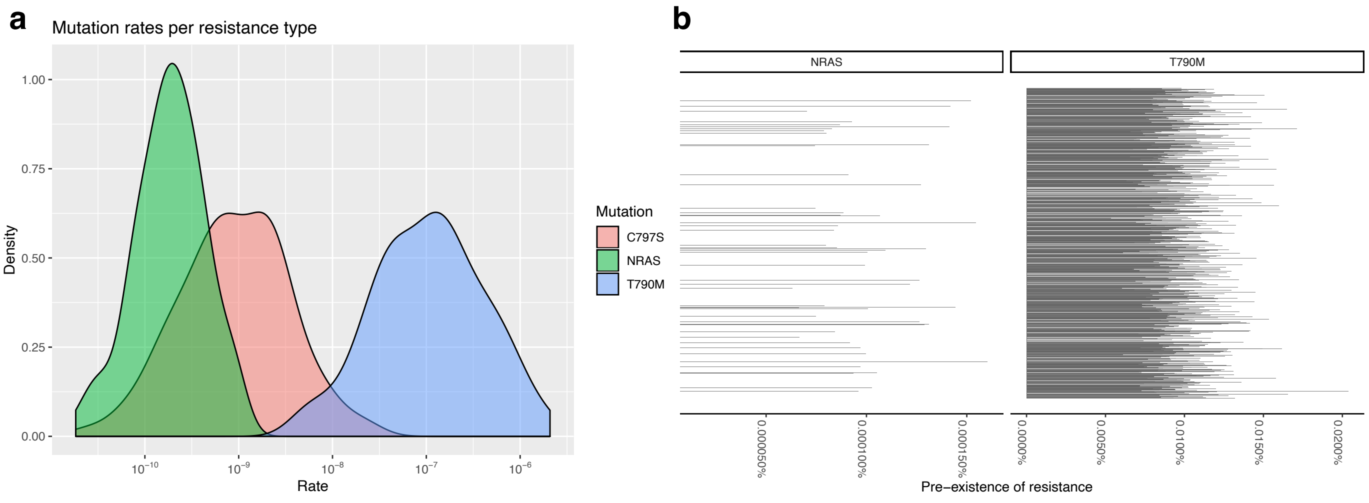

Fig S10. (a) Relative improvement percentage after 8 weeks of treatment by cell type. T790M clones are best suppressed in most patients, whereas there is less difference in exon 19 del. (b) Relative improvement percentage for patients with a weight of 90 kg, almost 30% above the reported median. Approximately 75% of patients would perform better with 30 mg dacomitinib QD and 40 mg osimertinib BID compared to 30 mg dacomitinib QD and 80 mg osimertinib QD, with a median improvement of 60%.

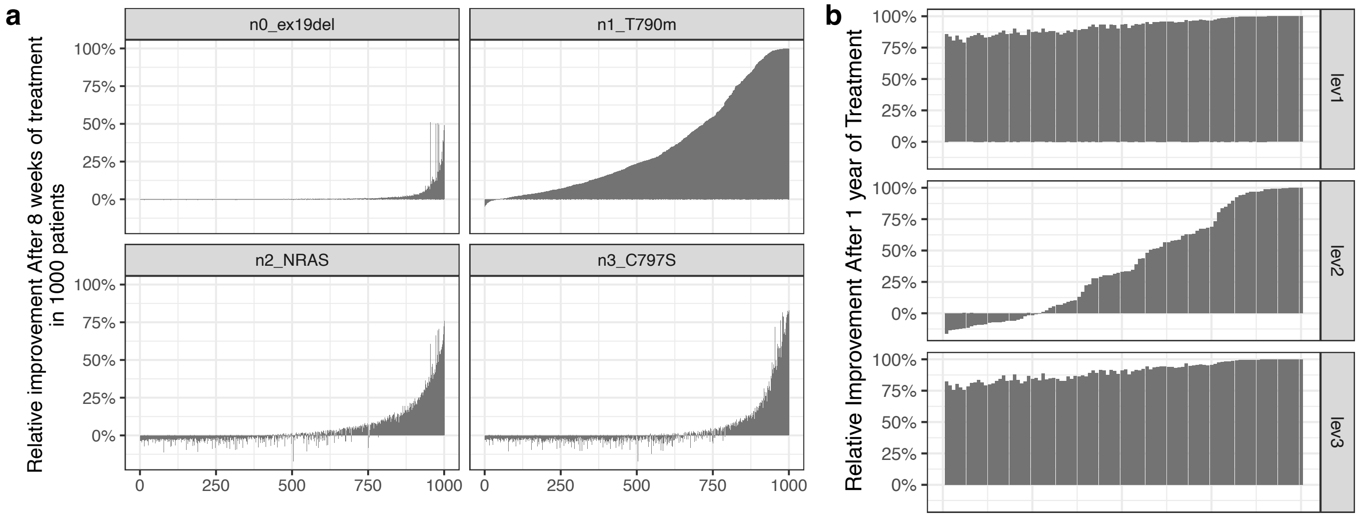

Fig S11. Random forest analysis to identify important variables. (a) Node purity of variables from random forest. The higher the purity, the more important the variable is when predicting improvement in outcomes. Results are shown for simulations administering two weeks of treatment. (b) Node purity of variables after a year of treatment. The pre-existence and prevalence of NRAS is no longer as important after a year of treatment than after two weeks. All random forests were run using 50 simulations, with 500 trees in each simulation. Mean node purity with standard errors are shown in black dots and error bars (n = 50 estimates), respectively. (c) Scatterplots of T790M+ cells against values of popPK parameters (these are constant across different doses and time). Clearance of both compartments from the osimertinib pop-PK model were highly correlated with predicted T790M+ cell count (Spearman correlation test with Bonferroni correction). (d) Scatterplots of C797S+ cells against values of popPK parameters. As expected, some PK parameters from the dacomitinib popPK model were significantly correlated with clone size. Parameter descriptions are included in **Table S4**.

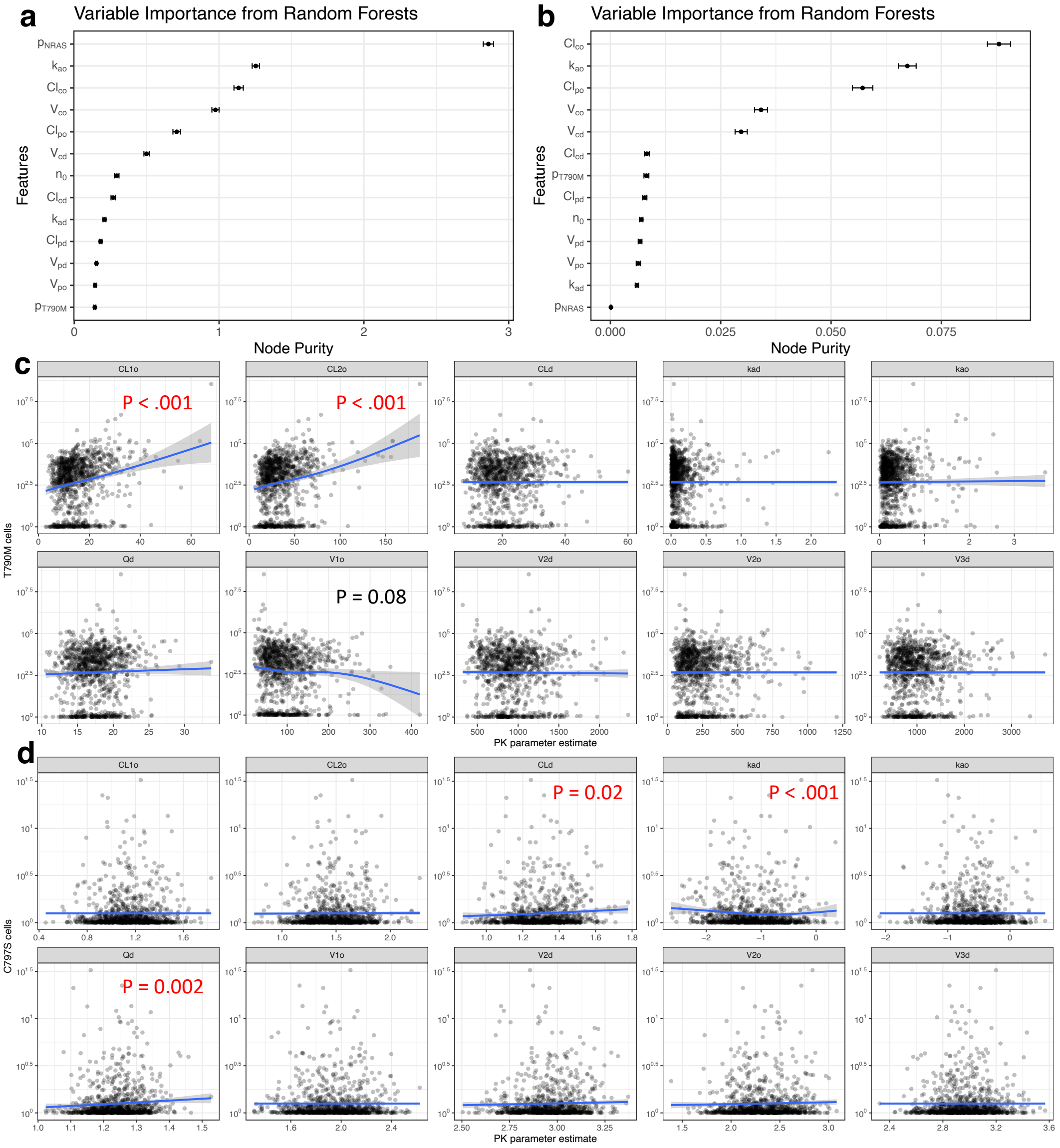

Fig S12. Validation experiment design and results in parent PC9 cell line. (a) Arms for DoR experiments. Each number is a drug concentration combination modeled after a dose combination. We did not use concentrations modeled from the level 2 schedules since conventional and proposed schedules yield the same concentration *in vitro*. (b) Validation of PC9 cell line from DoR experiments. Points and error bars (n=3 biological replicates for each condition) represent mean and standard deviation of cell counts, respectively, and and dashed line is median cell counts (with shaded IQR) from computational simulations. Like other long-term assays, our model overestimated cell count past 60 days in many schedules. (c) Validation of RPC9-CL6 cell line (90% exon 19 del and 10% exon 19 del+T790M) on day 10 and 20, our predictions correctly ranked the schedules for this cell pool. Mean and standard error of observations are shown in black dots and error bars (n=3 biological replicates for each condition), respectively. Predictions and interquartile ranges are shown in red dashed lines and shaded regions, respectively. (d) The DOR studies for PC9-NRAS only lasted 10 days, and all schedules seemed to perform equivalently on day 10 as predicted. Mean and standard error of observations are shown in black dots and error bars (n=3 biological replicates for each condition), respectively. Predictions and interquartile ranges are shown in red dashed lines and shaded regions, respectively.

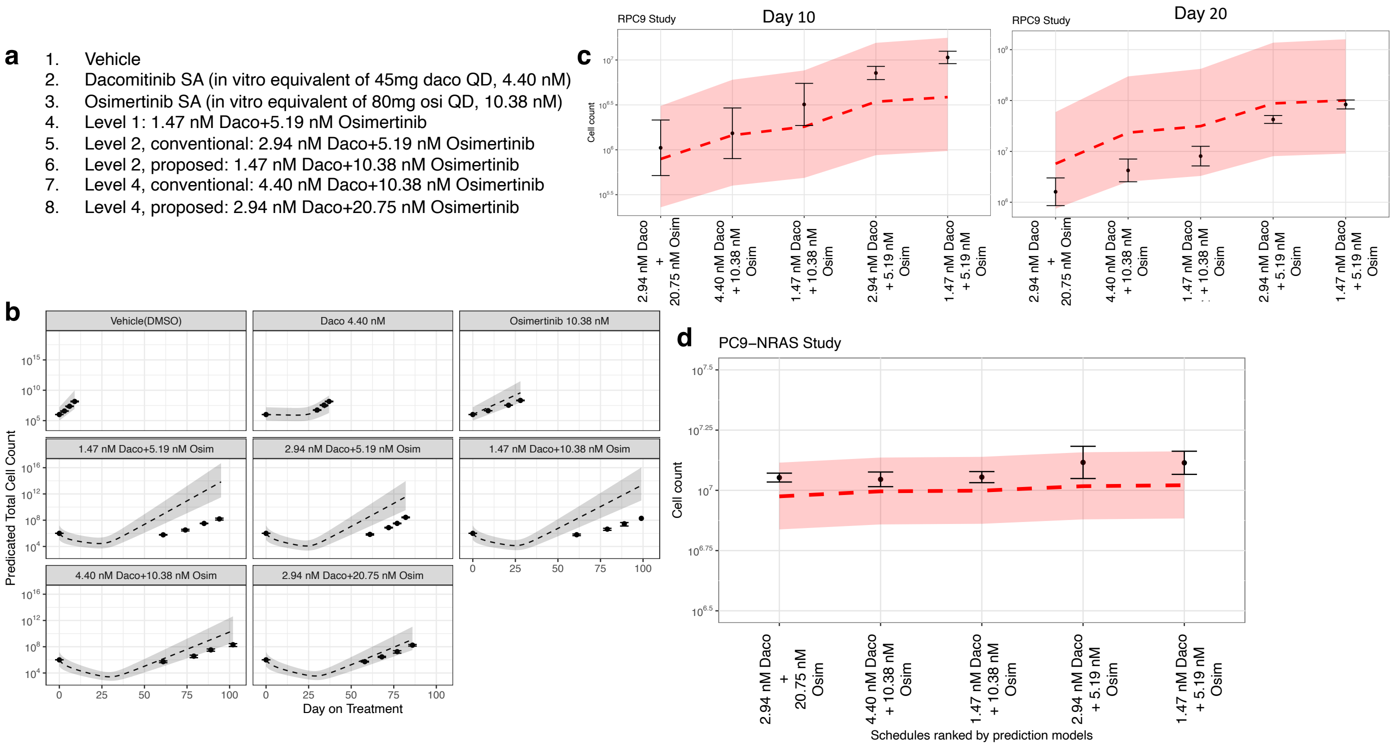

Table S1: Estimated growth rate constants (h^{-1} units) and standard errors (SE) by drug concentration. These estimates are taken from the CTG experiments and represent the slopes from figures 1C and S2. The outcome, tumor cell count, was log-transformed and regressed against time (in hours) via ordinary least squares.

Daco (nM)	Osi (nM)	PC9	SE of PC9	PC9-T790M	SE of PC9-T790M	PC9-C797S	SE of PC9-C797S	PC9-NRAS	SE of PC9-NRAS
**0**	**0**	0.0246	4e-04	0.0158	0.0011	0.017	0.0022	0.0211	0.0021
**0**	**0.017**	0.0237	4e-04	0.0168	0.0026	0.0136	0.0023	0.0242	0.0019
**0**	**0.051**	0.0242	7e-04	0.0161	0.0026	0.014	0.0015	0.0238	0.0021
**0**	**0.152**	0.0239	4e-04	0.016	0.0024	0.0098	0.0024	0.0236	0.0026
**0**	**0.457**	0.0232	4e-04	0.0158	0.0024	0.0166	0.0023	0.024	0.0024
**0**	**1.372**	0.0231	5e-04	0.0162	0.0025	0.0142	0.002	0.0236	0.003
**0**	**4.115**	0.0183	5e-04	0.0162	0.0021	0.0102	0.0026	0.0227	0.0033
**0**	**12.35**	0.0058	7e-04	0.0146	0.0022	0.0117	0.0021	0.0224	0.0039
**0**	**37.04**	-0.0017	0.001	0.0078	8e-04	0.0138	0.002	0.0226	0.0042
**0**	**111.1**	-0.0043	9e-04	-0.0035	0.0012	0.0121	0.0011	0.0215	0.0047
**0**	**333.3**	-0.0035	0.001	-0.0126	5e-04	0.0132	7e-04	0.0214	0.0047
**0**	**1000**	-0.004	0.001	-0.0131	8e-04	0.0093	0.0029	0.0194	0.0028
**0.004**	**0**	0.025	8e-04	0.0164	0.0012	0.015	0.0027	0.019	0.0027
**0.013**	**0**	0.0243	0.0011	0.0151	0.0017	0.0173	0.0034	0.0194	0.0025
**0.038**	**0**	0.0242	9e-04	0.0152	0.002	0.0149	0.002	0.0197	0.0026
**0.114**	**0**	0.0237	0.001	0.0148	0.0019	0.0151	0.0047	0.019	0.0026
**0.343**	**0**	0.0249	0.0018	0.0143	0.0019	0.0187	0.0043	0.0189	0.0023
**1.029**	**0**	0.005	6e-04	0.0146	0.0023	0.0179	0.0031	0.0191	0.0019
**3.086**	**0**	-0.0028	0.0013	0.0138	0.0022	0.0153	0.0044	0.0195	0.0024
**9.259**	**0**	-0.0041	0.0014	0.0138	0.0024	0.0091	0.0031	0.0194	0.0018
**27.78**	**0**	-0.0048	0.0012	0.0143	0.0026	-0.0062	0.0034	0.0196	0.002
**83.33**	**0**	-0.0049	0.0016	0.0136	0.0028	-0.0084	0.0041	0.0192	0.0016
**250**	**0**	-0.0056	0.0019	0.0136	0.0014	-0.01	0.0031	0.0198	9e-04

Table S2. Symptoms causing dose reduction and discontinuation in patients treated with osimertinib and dacomitinib.

Symptom	N
**Dose reduction**	
Gr. 1-2 Rash	5
Gr. 1-2 Mucositis	3
Gr. 1-2 Diarrhea	3
Gr. 3 Diarrhea	2
Gr. 1 Pain of skin	1
Gr. 2. Urticaria	1
Gr. 2 Rectal pain, hemorrhoids	1
Gr. 2 Lung infection	1
Gr. 2 Anorexia	3
Gr. 3 Dry Skin	1
Gr. 3 Prolonged QT	1
Gr. 3 Weight Loss	1
Gr. 2 Onychoschizia	1
**Study Discontinuation**	
Gr. 2 diarrhea and Gr. 1 hemorrhoidal hemorrhage	1
Gr. 1 Diarrhea, nausea, fatigue, anorexia	1
Gr. 2 Anorexia and Fatigue	1

Table S3. List of antibodies with dilution used in the immunoblotting analysis. All antibodies were purchased from Cell Signaling Technology® and have been validated in published studies listed below.

**Antibody (Product No)**	**Dilution**	**Validation**
EGFR (4267)	1:1000	PMID: 12063263
p-EGFR Y1068 (3777)	1:1000	PMID: 12063263
ERK (9102)	1:1000	PMID: 9038193
p-ERK T202/Y204 (9101)	1:1000	PMID: 8622663
Akt (4691)	1:1000	PMID: 10864894
pAktS473 (4060)	1:2000	PMID: 10864894
S6 ribosomal protein (2217)	1:1000	PMID: 15960972
pS6 (4858)	1:2000	PMID: 19029981
cleaved PARP (9541)	1:1000	PMID: 11154281
GAPDH (2118)	1:2000	PMID: 17488287

Table S4. Description of popPK parameters from Fig S11.

Parameter	Description
Cl_{co} CL1o	Clearance of central compartment (osimertinib)
Cl_{po}, CL2o	Clearance of peripheral compartment (osimertinib)
Cl_{cd}, CLd	Clearance of central compartment (dacomitinib)
k_{ad}, kad	Absorption to central compartment (dacomitinib)
k_{ao}, kao	Absorption to central compartment (osimertinib)
Cl_{pd}, Qd	Clearance of peripheral compartment (dacomitinib)
V_{co}, V1o	Volume distribution of central compartment (osimertinib)
V_{cd}, V2d	Volume distribution of central compartment (dacomitinib)
V_{po}, V2o	Volume distribution of peripheral compartment (osimertinib)
V_{pd}, V3d	Volume distribution of peripheral compartment (dacomitinib)

Phase 1 Study of Combination Dacomitinib and Osimertinib for Patients with Metastatic EGFR Mutant Lung Cancers

PROTOCOL FACE PAGE FOR
MSK THERAPEUTIC/DIAGNOSTIC PROTOCOL

Principal Investigator/Department:	Helena Yu, MD	Medicine
Co-Principal Investigator(s)/Department:	Gregory Riely, MD, PhD	Medicine
Investigator(s)/Department:	Charles Rudin, MD, PhD<br>Alexander Drilon, MD<br>Matthew Hellmann, MD<br>Paul Paik, MD<br>Mark Kris, MD<br>Piro Lito, MD, PhD<br>Jamie Chaft, MD<br>Marjorie Zauderer, MD<br>Stephen Veach, MD<br>Bob Li, MD<br>Robert Daly, MD, MBA<br>Katherine Arbour, MD<br>Victoria Lai, MD<br>Michael Offin, MD<br>Arlyn Apollo, MD<br>Pamela Drullinksy, MD<br>Zoe Goldberg, MD<br>Oscar Lahoud, MD<br>Kenneth Ng, MD<br>Tiffany Troso-Sandoval, MD<br>Avni Desai, MD<br>Juliana Eng, MD<br>John Fiore, MD<br>Jia Li, MD<br>Sree Chalasani, MD<br>Afsheen Iqbal, MD<br>Loren Michel, MD<br>Han Xiao, MD<br>Colette Owens, MD<br>Azadeh Namakydoust, MD<br>Marina Shcherba, DO<br>Daniel Danila, MD<br>Ping Gu, MD<br>Anuja Kriplani, MD<br>Louise Ligresti, MD<br>Isabel Preeshagul, DO<br>Rui Wang, MD PhD<br>Alice Zervoudakis, MD<br>James Fetten, MD<br>Parisa Momtaz, MD	Medicine<br>Medicine

	Matthew Matasar, MDLinda Ahn, NP<br>Elizabeth Panora, NP<br>Alison Massey, NP<br>Christina Larsen, NP<br>Erica Stumpf, NP<br>Diana Velez, NP<br>Leticia Smith, APN<br>Geri Arnell, RN<br>Joanne Wells, APN<br>Janet Cogswell, RN<br>Megan Stasi, RN<br>Ofer Maimran, RN<br>Nicole Heinz, RN<br>Raylene Langish, RN<br>Jessica Marchisotto, RN<br>Karen Flynn, APN<br>Irene Kelly, RN<br>Sherie Mar-Chaim, RN<br>Gloria Wasilewski, RN<br>Maureen Kennedy, RN<br><br>Glenn Heller, MD<br><br>Sara Hayes, MD<br>Robert Young, MD<br>Andrew Plodkowski, MD	Medicine<br>Nursing<br><br>Epidemiology/Biostatistics<br><br>Radiology<br>Radiology<br>Radiology
Consenting Professional(s)/Department:	Helena Yu, MD<br>Mark Kris, MD<br>Charles Rudin, MD, PhD<br>Alexander Drilon, MD<br>Matthew Hellmann, MD<br>Paul Paik, MD<br>Gregory Riely, MD, PhD<br>Piro Lito, MD, PhD<br>Jamie Chaft, MD<br>Marjorie Zauderer, MD<br>Stephen Veach, MD<br>Bob Li, MD<br>Robert Daly, MD, MBA<br>Kathryn Arbour, MD<br>Victoria Lai, MD<br>Michael Offin, MD<br>Arlyn Apollo, MD<br>Pamela Drullinksy, MD<br>Zoe Goldberg, MD<br>Oscar Lahoud, MD<br>Kenneth Ng, MD<br>Tiffany Troso-Sandoval, MD<br>Avni Desai, MD<br>Juliana Eng, MD<br>John Fiore, MD<br>Jia Li, MD	Medicine<br>Medicine

	Sree Chalasani, MD<br>Afsheen Iqbal, MD<br>Loren Michel, MD<br>Han Xiao, MD<br>Colette Owens, MD<br>Azadeh Namakydoust, MD<br>Marina Shcherba, DO<br>Daniel Danila, MD<br>Ping Gu, MD<br>Anuja Kriplani, MD<br>Louise Ligresti, MD<br>Isabel Preeshagul, DO<br>Rui Wang, MD PhD<br>Alice Zervoudakis, MD<br>James Fetten, MD<br>Parisa Momtaz, MD<br>Matthew Matasar, MD	Medicine<br>Medicine<br>Medicine<br>Medicine<br>Medicine<br>Medicine<br>Medicine<br>Medicine<br>Medicine<br>Medicine<br>Medicine<br>Medicine<br>Medicine<br>Medicine<br>Medicine<br>Medicine<br>Medicine

Please Note: A Consenting Professional must have completed the mandatory Human Subjects Education and Certification Program.

OneMSK Sites	
Manhattan	All protocol activites
Basking Ridge	All protocol activites
Bergen	All protocol activites
Commack	All protocol activites
Monmouth	All protocol activites
Westchester	All protocol activites
Nassau	All protocol activites

Memorial Sloan Kettering Cancer Center
1275 York Avenue
New York, New York 10065

Table of Contents

1.0 PROTOCOL SUMMARY AND/OR SCHEMA 5
2.0 OBJECTIVES AND SCIENTIFIC AIMS 8
3.0 BACKGROUND AND RATIONALE 8
4.0 OVERVIEW OF STUDY DESIGN/INTERVENTION 12
4.1 Design 12
4.2 Intervention 12
5.0 THERAPEUTIC/DIAGNOSTIC AGENTS 13
6.0 CRITERIA FOR SUBJECT ELIGIBILITY 14
6.1 Subject Inclusion Criteria 14
6.2 Subject Exclusion Criteria 14
7.0 RECRUITMENT PLAN 15
8.0 PRETREATMENT EVALUATION 15
9.0 TREATMENT/INTERVENTION PLAN 16
10.0 EVALUATION DURING TREATMENT/INTERVENTION 17
11.0 TOXICITIES/SIDE EFFECTS 18
12.0 CRITERIA FOR THERAPEUTIC RESPONSE/OUTCOME ASSESSMENT 23
13.0 CRITERIA FOR REMOVAL FROM STUDY 25
14.0 BIOSTATISTICS 25
15.0 RESEARCH PARTICIPANT REGISTRATION AND RANDOMIZATION PROCEDURES 28
15.1 Research Participant Registration 28
16.0 DATA MANAGEMENT ISSUES 29

16.1 Quality Assurance 29
16.2 Data and Safety Monitoring 29
17.0 PROTECTION OF HUMAN SUBJECTS 30
17.1 Privacy 31
17.2 Serious Adverse Event (SAE) Reporting 31
18.0 INFORMED CONSENT PROCEDURES 32
19.0 REFERENCES 33

1.0 PROTOCOL SUMMARY AND/OR SCHEMA

**Study Title:**	Phase 1 Study of Combination Dacomitinib and Osimertinib for Patients with Metastatic EGFR Mutant Lung Cancers
**Study Objectives:**	**Phase 1 Escalation Cohort:**<br>Primary Objective: To determine maximum tolerated dose of the combination of dacomitinib and osimertinib in patients with *EGFR* mutated advanced non-small cell lung cancer (NSCLC).<br><br>Secondary Objectives: 1) measure best overall response, 2) measure progression-free survival (PFS) 3) measure overall survival (OS) 4) determine the frequency of EGFR T790M and C797S emergence on this combination regimen.<br>**Phase 1 Expansion Cohort:**<br>Primary objective: to further establish the toxicity profile of the combination of combination dacomitinib and osimertinib.<br><br>Secondary objective: to obtain preliminary efficacy data by measuring the objective response rate (sum of complete responses and partial responses according to RECIST 1.1), progression free survival and overall survival.<br><br>**Correlative Studies:**<br>Objectives: 1) identify any pre-treatment biomarkers that predict response to combination osimertinib/dacomitinib in this setting 2) identify molecular biomarkers within plasma (sensitizing EGFR mutation, EGFR C797S, EGFR T790M), quantify and follow serially over time. 3) determine the mechanisms of resistance to osimertinib/dacomitinib treatment
**Patient Population:**	Patients with EGFR-mutant metastatic lung adenocarcinomas without prior EGFR TKI treatment
**Number of patients:**	Phase 1: 2-34 patients (up to 34 patients)

**Inclusion Criteria:**	All patients must have:<br>• Written informed consent<br>• Advanced biopsy-proven metastatic non-small cell lung cancer<br>• Somatic activating mutation in EGFR in pre-treatment biopsy<br>• No prior standard or experimental EGFR inhibitor treatment (osimertinib, dacomitinib, gefitinib, afatinib, erlotinib)<br>• Archival tissue available from a tumor biopsy or willing to undergo a tumor biopsy prior to study initiation.<br>• Measurable (RECIST 1.1) indicator lesion not previously irradiated<br>• Karnofsky performance status (KPS) ≥ 70%<br>• Age >18 years old<br>• Adequate organ function<br>• Ability to swallow oral medications
**Exclusion Criteria:**	Patients are to be excluded from the study if they meet any of the following criteria:<br>• Symptomatic brain metastases or leptomeningeal disease requiring escalating doses of steroids<br>• Pregnant or lactating women<br>• Any radiotherapy within 1 week of starting treatment on protocol.<br>• Any major surgery within 1 week of starting treatment on protocol.<br>• Any evidence of clinically significant interstitial lung disease<br>• Continue to have unresolved > CTCAE grade 1 toxicity from any previous treatment
**Study drug:**	Dacomitinib, Osimertinib

Design

Phase 1: The study will assess whether combination dacomitinib and osimertinib is safe and tolerable in patients with metastatic EGFR-mutant lung cancers. There are four pre-planned dose-levels with escalating does of osimertinib and dacomitinib. The doses of dacomitinib and osimertinib were derived by modeling various dosing schedules to find the optimal dose combination. The model considers pharmacokinetic (PK) properties of drugs, growth rate of tumor at different treatment levels, toxicity of drugs and includes inter-subject variability in drug absorption and elimination.

Modeling derived dose levels:

Dose level	Dacomitinib	Osimertinib
Dose level 1	Dacomitinib 15mg daily	Osimertinib 40mg daily
Dose level 2	Dacomitinib 15mg daily	Osimertinib 40mg BID
Dose level 3	Dacomitinib 30mg daily	Osimertinib 40mg BID

Patients will begin on combination dacomitinib and osimertinib at the prescribed doses. A cycle will be 28 days in duration. The study will use a standard 3+3 dose escalation design. Three to six patients will need to be enrolled at each dose level and assessed for DLT for 1 full cycle (28 days for cycle 1) before dose escalation decision is made. Once the MTD is established, there will be an expansion cohort of an additional 10 patients treated at the recommended phase 2 dose, likely the MTD, to further assess safety and preliminary efficacy.

Toxicity will be graded according to NCI CTCAE version 5. Response to therapy will be assessed with serial CT imaging every 2 cycles with CT scan of the chest/abdomen/pelvis with response evaluated by RECIST 1.1. Dose reductions after the initial DLT period can be made and are detailed in Section 11.0. Intra-patient dose-escalation will be permitted and is detailed in the protocol.

Correlative studies: IMPACT will be done on archival tissue obtained before treatment on protocol. We will note if any molecular findings (C797S, EGFR amplification, HER2 amplification, etc) are associated with response to combination therapy. In addition, at screening, every 2 cycles and at disease progression, we will obtain a plasma sample for molecular testing. Allele frequency of specific EGFR mutations (sensitizing EGFR mutation, T790M, C797S) will be quantified and followed over time. A rebiopsy at the time of clinical progression on EGFR TKI will not be mandated per protocol (optional) but is typically done as per standard of care. We will collect molecular data from this repeat biopsy and attempt to identify potential mechanisms of resistance to combination dacomitinib/osimertinib therapy.

Time to completion:

Over an 18 month period (8/2016-1/2018), we enrolled 44 patients on to a first line osimertinib and bevacizumab study (16-033). We also get a large number of referrals for clinical trials for patients with EGFR-mutant lung cancer. We will enroll 2-3 patients a month and complete enrollment in 1.5 years.

2.0 OBJECTIVES AND SCIENTIFIC AIMS

Phase 1 Escalation Cohort:
Primary Objective: To determine maximum tolerated dose of the combination of dacomitinib and osimertinib in patients with *EGFR* mutated advanced non-small cell lung cancer (NSCLC).

Secondary Objectives: 1) measure best overall response, 2) measure progression-free survival (PFS) 3) measure overall survival (OS) 4) determine the frequency of EGFR T790M and C797S emergence on this combination regimen.

Phase 1 Expansion Cohort:
Primary objective: to further establish the toxicity profile of the combination of combination dacomitinib and osimertinib.

Secondary objective: to obtain preliminary efficacy data by measuring the objective response rate (sum of complete responses and partial responses according to RECIST 1.1), progression free survival and overall survival.

Correlative Studies:
Objectives: 1) identify any pre-treatment biomarkers that predict response to combination osimertinib/dacomitinib in this setting 2) identify molecular biomarkers within plasma (sensitizing EGFR mutation, EGFR C797S, EGFR T790M), quantify and follow serially over time. 3) determine the mechanisms of resistance to osimertinib/dacomitinib treatment

3.0 BACKGROUND AND RATIONALE

EGFR-mutant lung cancers
Twenty percent of patients with metastatic lung adenocarcinoma have somatic activating mutations in the epidermal growth factor receptor gene (EGFR). Patients with EGFR-mutant lung adenocarcinomas have a 70% response rate to first-line earlier generation EGFR-TKI therapy (erlotinib or afatinib) and a median progression-free survival of 9-12 months [3]. First-line EGFR TKI therapy is superior to cytotoxic chemotherapy and is recommended as first-line treatment for patients with EGFR-mutant lung cancers.

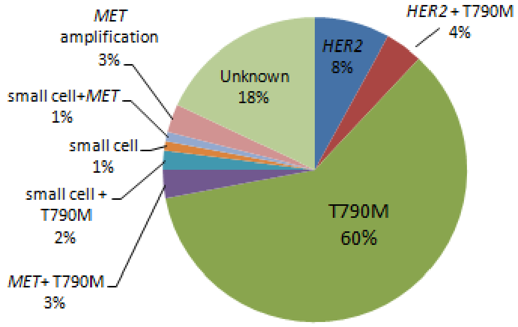

Figure 1

Dacomitinib

Dacomitinib is a pan-HER tyrosine kinase inhibitor that has demonstrated efficacy as first-line treatment for patients with EGFR-mutant lung cancers. In ARCHER 1050, dacomitinib was compared to gefitinib as first-line treatment for patients with EGFR-mutant lung cancers[4]. The median progression-free survival by blinded independent review (the study primary endpoint) was 14.7 months for dacomitinib compared to 9.2 months for gefitinib demonstrating impressive efficacy compared to the current standard of care EGFR TKI. Dacomitinib as of September 2018 has been approved for first line treatment for patients with metastatic EGFR-mutant lung cancers.

Figure 2: Mechansms of resistance to EGFR TKI[2]

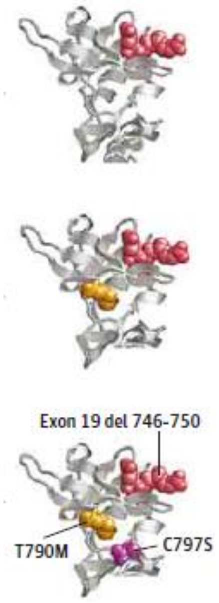

Mechanisms of resistance to earlier generation EGFR TKIs
EGFR TKIs, although initially effective, induce incomplete tumor responses followed by drug resistance and clinical disease progression. Resistance mechanisms to EGFR TKIs are identified on rebiopsy at the time of clinical progression and include acquired EGFR T790M mutations, MET amplification, HER2 amplification and small cell histologic transformation (Figure 2) [2]. These resistance mechanisms are critical to identify because they inform subsequent treatment decisions. EGFR T790M is acquired in 60% of patients with EGFR-mutant lung cancers on EGFR TKIs and confers sensitivity to EGFR T790M inhibitors such as osimertinib.

Osimertinib
Osimertinib is FDA approved for the treatment EGFR-mutant lung cancers that have progressed on initial EGFR TKI and harbor EGFR T790M. The overall response rate to Osimertinib in patients who harbor EGFR T790M in their lung cancers after progression on EGFR TKI is 71% compared to 31% with platinum plus pemetrexed chemotherapy[5]. Due to its efficacy in the second line setting, osimertinib was evaluated as first line therapy for patients with EGFR mutant lung cancer in the FLAURA study [6]. In the FLAURA study, patients were randomized to receive either osimertinib or standard of care EGFR TKI (erlotinib or gefitinib) as first-line treatment.The median progression-free survival was longer with osimertinib compared to standard EGFR TKI (gefitinib or erlotinib), 18.9 months compared to 10.2 months. This study has resulted in the inclusion of first-line treatment with osimertinib into the NCCN guidelines and recent FDA approval of osimertinib (April 2018) as a first-line treatment for patients with EGFR-mutant lung cancers.

Osimertinib 160mg daily has been assess in the BLOOM study as well as a subset of the AURA study [14, 15]. Osimertinib 160mg daily showed similar toxicity profile with osimertinib 80mg daily. Related serious adverse events that were related to study treatment were lower in the 160mg group (n=30) compared to 80mg (n-30), 3% vs 13%. Adverse events leading to discontinuation of osimertinib was lower in the 160mg group compared to 80mg, 3% vs 7%. Adverse events leading to dose reduction was higher in the osimertinib 160mg group compared to 80mg, 53% vs 10%.

Mechanisms of resistance to osimertinib
Similar to the other available EGFR TKIs, responses to osimertinib are limited with eventual disease progression. There is emerging data as to what the mechanisms of resistance to osimertinib may be, although the majority of the data are from 2nd line and later osimertinib treatment. In up to 35% of cases, there is evidence of an acquired EGFR C797S mutation[7] (Figure 2). Similar to EGFR T790M and earlier generation EGFR TKIs, osimertinib binds to EGFR at C797, so a mutation at this location prohibits drug binding and results in loss of drug efficacy. Interestingly, in the presence of the original EGFR activating mutation (ex19del or L858R) and EGFR C797S without EGFR T790M, cells retain sensitivity to first and second generation EGFR TKIs[8]. First-line osimertinib treatment does not appear to lead to acquired EGFR T790M. Other non-EGFR mediated resistance mechanisms to osimertinib have also been identified including amplification of MET, EGFR and HER2, as well as acquired mutations in PIK3CA, BRAF, and KRAS.

Evolutionary modeling

Use of mathematical models for treatment optimization is part of a growing effort to improve clinical trial design for cancer patients. These models can investigate the relationship between toxicity and dose, but also can utilize in vitro data to help understand the relationship between efficacy and exposure. This computational modeling can incorporate both in vitro data and published clinical data to investigate the efficacy of combination dacomitinib and osimertinib as a function of different dosing schedules to best inhibit EGFR mutant cell growth. The goal is to find the best dosing schedule balancing toxicity and efficacy while considering pharmacokinetic (PK) properties of drugs, growth rate of tumor at different treatment levels, toxicity of drugs and inter-subject variability in drug absorption and elimination. Dr. Michor and our group have collaborated on this previously [9] and she has extensive experience with optimizing targeted therapy dosing and in different tumor types [10, 11].

Model of combination dacomitinib and osimertinib treatment

The model we used for this proposal consists of combining 3 approaches: tumor evolution and growth under different dosing schedules, population pharmacokinetic (popPK) models of drugs, and toxicity constraints. We performed *in silico* clinical trials and assessed the most effective dosing schedule by comparing expected number of tumor cells after 21 days of treatment.

Our approaches, however, operate on a few assumptions mentioned below. When modeling tumor evolution and growth, we assume that tumor cells follow an exponential growth. In other words, one parent cell gives rise to two daughter cells and each daughter cell gives rise to two more cells, and so on. We analyze two type of tumor cells, a sensitive cell and resistant cell, the latter being a descendant from the sensitive cell that has acquired a resistant mechanism from the present treatment. Additionally, we suppose that the mutation rate is 10^{-7} mutation/bp/generation, an approximate estimate for the probability of a base pair being substituted[12]. In this model, each type of tumor cell has its own growth proliferation rate. We obtain PC9 cell counts from imaging data from IncuCyte and estimate a growth proliferation rate at different treatment levels. We regress growth rates against transformed values of drug concentrations to determine drug effect on tumor cells and predict future growth proliferation rates. We presume that dacomitinib and osimertinib do not have a synergistic nor antagonistic interaction.

For the popPK models, we use prior literature and an unpublished study from Pfizer. We assume a first-order elimination rate in both drugs which are administered orally. Additional assumptions for osimertinib have been published[13]. As for dacomitinib, we assume it follows a two compartment model, and is affected by various factors including sex, race, albumin, and EGFR mutation status. In addition, inter-individual variability for each parameter follows a log-normal distribution. When consolidating both popPK models, we speculate that intra-subject correlation between the drugs' random effects is from 0.3 to 0.7, depending on which PK parameter is being used. In summary, we use these models to simulate drug concentrations in plasma for patients with specific characteristics. Supposing our assumptions above are correct, our model indicates that the proposed schedules will yield the optimal results from any other combination of drugs within the constraints of toxicity of combining two EGFR TKIs. An interesting consequence that we observed was that dosing schedules with higher/more frequent osimertinib doses and lower (or less frequent) dacomitnib doses performed better than what have been a standard combination dosing dose escalation plan (group 1 in the figure below). We observed a median relative improvement of 81%, 26%, and 15% from dose level -1 to dose level 1, level 1 to level 2, level 2 to level 3, respectively.

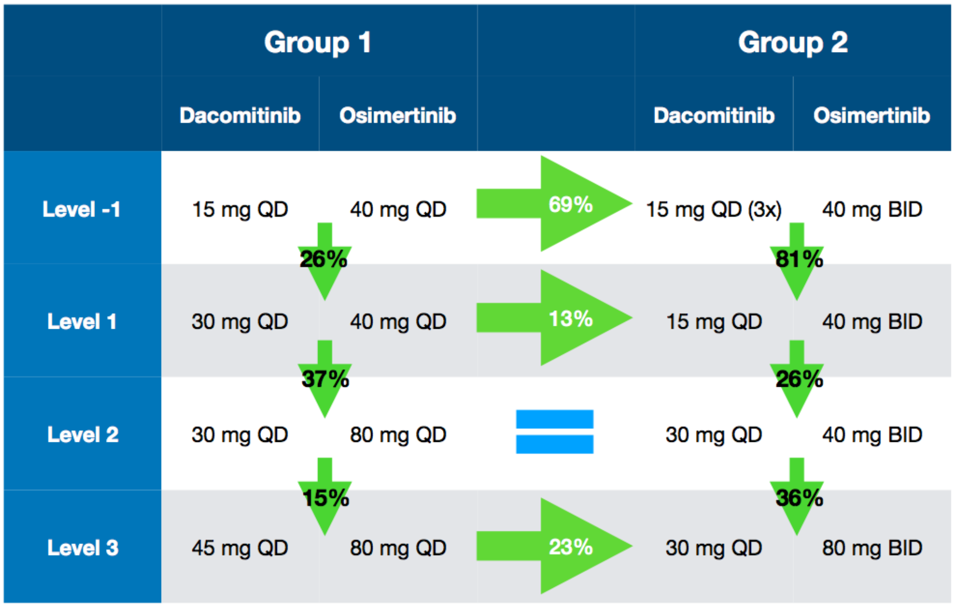

Figure 3: Median relative improvements in cell death with different dosing schedules of dacomitinib and osimertinib. Group 1 was what would be considered a standard combination dose escalation that culminates (dose level 3) in full doses of both drugs. Group 2 contains our proposed dosing schedules. There is an improvement at each level from group 1 to group 2. Note that level 2 of both groups have the same effect (and are essentially equal doses of both drugs).

Using our model, we identified combination dacomitinib and osimertinib doses that optimize efficacy but stay within the contrainsts of toxicity of combining two EGFR TKIs.

Table 3-1: Modeling derived dose levels:

Dose level	Dacomitinib	Osimertinib
Dose level 1	Dacomitinib 15mg daily	Osimertinib 40mg daily
Dose level 2	Dacomitinib 15mg daily	Osimertinib 40mg BID
Dose level 3	Dacomitinib 30mg daily	Osimertinib 40mg BID

<u>Study rationale/Innovation</u>:

Osimertinib is now approved as first-line treatment for patients with metastatic EGFR-mutant lung cancer. There are currently no EGFR targeted therapies that have demonstrated efficacy after progression on osimertinib. Dacomitinib is a pan-HER inhibitor with demonstrated superiority over other earlier generation EGFR TKIs as first-line treatment for EGFR-mutant lung cancers. In the setting of osimertinib treatment and subsequent acquired EGFR C797S, earlier generation EGFR inhibitors such as dacomitinib, have demonstrated pre-clinical efficacy. In the setting of dacomitinib treatment and subsequent acquired EGFR T790M, osimertinib has demonstrated efficacy and is approved for treatment in that second line setting. In addition, dacomitinib may be able to address resistance to osimertinib driven by HER2 or EGFR amplification as well. Mathematical modeling is a novel means to optimize dosing of combination targeted therapy to optimize efficacy while minimizing toxicity which may be especially important for targeted therapies where the efficacious dosing range is wide but may also be limited by toxicity. For these reasons, we have proposed a phase 1 dose escalation study of modeling derived dose levels of combination dacomitinib and osimertinib as first line treatment for patients with EGFR-mutant lung cancer.

4.0 OVERVIEW OF STUDY DESIGN/INTERVENTION

4.1 Design

Phase 1: The study will assess whether combination dacomitinib and osimertinib is safe and tolerable in patients with metastatic EGFR-mutant lung cancers. There are four pre-planned dose-levels with escalating does of osimertinib and dacomitinib. The doses of dacomitinib and osimertinib were derived by modeling various dosing schedules to find the optimal dose combination. The model considers pharmacokinetic (PK) properties of drugs, growth rate of tumor at different treatment levels, toxicity of drugs and includes inter-subject variability in drug absorption and elimination.

Table 4-1: Modeling derived dose levels:

Dose level	Dacomitinib	Osimertinib
Dose level 1	Dacomitinib daily	Osimertinib 40mg daily
Dose level 2	Dacomitinib 15mg daily	Osimertinib 40mg BID
Dose level 3	Dacomitinib 30mg daily	Osimertinib 40mg BID

Patients will begin on combination dacomitinib and osimertinib at the prescribed doses. A cycle will be 28 days in duration. The study will use a standard 3+3 dose escalation design. Three patients will need to be enrolled at each dose level and assessed for DLT for 1 full cycle (28 days for cycle 1) before dose escalation decision is made. Please see section 14.0 for dose escalation rules. 6 patients will need to be treated at a dose level for it to be determined the maximum tolerated dose (MTD). Once the MTD is established, there will be an expansion cohort of an additional 10 patients treated at the recommended phase 2 dose, likely the MTD, to further assess safety and preliminary efficacy. Over time, if there are intolerable grade 1-2 toxicities that emerge necessitating dose reduction in >25% of subjects, we may consider a dose lower than the MTD as the recommended phase 2 dose.

Toxicity will be graded according to NCI CTCAE version 5. Response to therapy will be assessed with serial CT imaging every 2 cycles with CT scan of the chest/abdomen/pelvis with response evaluated by RECIST 1.1. Dose reductions after the initial DLT period can be made and are detailed in Section 11.0. Intra-patient dose-escalation will be permitted and detailed in Section 9.0

4.1.1 Determining the Maximum Tolerated Dose (MTD)

Before the MTD can be determinded, 6 patients will need to be treated at a dose level, and 1 or less of those patients may experience a dose limiting toxicity (DLT). If 2 patients at a dose level experience a DLT, the previous dose level that was determined to be safe will be deemed the MTD. In the event that the first dose level in the study is determined to be too toxic, the trial will be stopped.

4.2 Intervention

4.2.1 Pre-treatment tumor sample collection

Tumor tissue will be collected from the biopsy prior to initiation of therapy on study. The equivalent of >10 unstained slides are typically required to obtain adequate DNA to perform MSK-IMPACT

testing. If patient has sufficient archival tissue, archival tissue can be utilized. In the subset of patients that do not have sufficient tissue, a repeat biopsy may be required.

4.2.2 Dacomitinib/Osimertinib therapy

Patient will be dosed with combination dacomitinib and osimertinib based on the dose level assigned.

Table 4-2:

Dose level	Dacomitinib	Osimertinib
Dose level 1	Dacomitinib 15mg daily	Osimertinib 40mg daily
Dose level 2	Dacomitinib 15mg daily	Osimertinib 40mg BID
Dose level 3	Dacomitinib 30mg daily	Osimertinib 40mg BID

Toxicity will be graded according to NCI CTCAE version 5. Toxicity (AEs experienced) and efficacy of the treatment for these patients will be recorded and presented in sum. Response to therapy will be assessed with serial CT imaging every 2 cycles with CT scan of the chest/abdomen/pelvis with response evaluated by RECIST 1.1.

4.2.3 Serial Plasma tumor collection

Plasma samples (2 streck tubes) will be obtained for cfDNA analysis. Plasma will be obtained during screening, at first folowup scan, and at the time of disease progression/end of treatment. ddPCR assessing for the various EGFR mutations- EGFR sensitizing mutation (L858R, exon 19 deletion, other), EGFR T790M and EGFR C797S will be performed. The different mutations will be identified, quantified and followed over time.

4.2.4 At progression tumor sample collection

At the time of disease progression on EGFR TKI therapy, it is standard of care for patients to proceed with a repeat tumor biopsy. All patients will undergo a separate clinical informed consent for any biopsies performed at MSK. All biopsy-related adverse events occurring on this study, in both the initial and subsequent biopsies, will be recorded on this study for analysis of the safety of biopsy in this context. We will aim to obtain up to 5 core samples for use as follows: surgical pathology for diagnosis (1 core), next-generation sequencing (2 cores) and PDX creation (2 cores). Any PDX creation will occur under IRB protocols 06-107, 12-245.

5.0 THERAPEUTIC/DIAGNOSTIC AGENTS

5.1 Dacomitinib

Dacomitinib will be provided by Pfizer Oncology. Dacomitinib is an investigational medication that is a white to pale colored powder that is provided as 15mg, 30mg or 45mg immediate release tablet using compendial excipients and a precedented film-coating. The tablets are packaged in appropriate packaging material including high density polyethylene (HDPE) bottles with dessicant, and will be stored as per labeled conditions. Dacomitinib is administered per the current dose level (irrespective of food intake). Concomitant use of proton pump inhibitors with dacomitinib should be avoided if possible, and if necessary, dosing timing should be spaced apart (morning and evening).

5.2 Osimertinib

Osimertinib will be commercially obtained. Osimertinib is a crystalline powder presented for oral administration as a beige, film- coated tablet containing either 40mg or 80mg of Osimertinib. Osimertinib beige film-coated tablets contain Osimertinib mesylate, mannitol, microcrystalline cellulose, low-substituted hydroxypropyl cellulose and sodium stearyl fumarate. Tablets will come in high-density polyethylene (HDPE) bottles with child-resistant closures. Tablets should be stored in their original packaging at room temperature. Osimertinib is administered as a once daily dose (irrespective of food intake).

6.0 CRITERIA FOR SUBJECT ELIGIBILITY

6.1 Subject Inclusion Criteria

- Written informed consent
- Advanced biopsy-proven metastatic non-small cell lung cancer
- Somatic activating mutation in EGFR in a tumor biopsy
- No prior EGFR inhibitor treatment (gefitinib, afatinib, erlotinib, dacomitinib, osimertinib) however, prior treatment with other chemotherapies are allowed
- Archival tissue available from a pre-treatment tumor biopsy or willing to undergo a tumor biopsy prior to study initiation.
- Measurable (RECIST 1.1) indicator lesion not previously irradiated
- Karnofsky performance status (KPS) ≥ 70%
- Age >18 years old
- Ability to swallow oral medication
- Agree to use effective methods of contraception from the time of screening until 3 months after treatment discontinuation (for males and females of child-bearing potential)
- Adequate organ function
  - AST, ALT ≤ 3 x ULN
  - Total bilirubin ≤ 1.5x ULN
  - Creatinine ≤ 1.5x ULN OR calculated creatinine clearance ≥ 60ml/min
  - Absolute neutrophil count (ANC) ≥ 1000 cells/mm^3
  - Hemoglobin≥9.0 g/dL
  - Platelets ≥100,000/mm^3

6.2 Subject Exclusion Criteria

- Pregnant or lactating women
- Any radiotherapy within 1 week of starting treatment on protocol.
- Any major surgery within 1 weeks of starting treatment on protocol.
- Any evidence of active clinically significant interstitial lung disease
- A mean QTc >470ms (Fridericia's correction), clinically important arrhythmia, conduction or morphology of resting ECG (eg complete LBBB, 1st -3rd degree heart block, any factors that increase the risk of QTc prolongation or risk of arrhythmia)
- Cardiovascular disease or cerebrovascular disease, CVA or MI < 6 months prior to study enrollment, unstable angina, NYHA >Grade II CHF, or serious cardiac arrhythmia uncontrolled by medication or with the potential to interfere with protocol treatment
- History of pneumonitis or interstitial lung disease (ILD), drug induced ILD, radiation pneumonitits that required steroid treatment, and any evidence of clinically active ILD
- Serious chronic GI conditions associated with diarrhea
- Symptomatic, unstable brain metastases requiring escalating doses of steroids

- Continue to have unresolved > CTCAE grade 1 toxicity from any previous treatment

7.0 RECRUITMENT PLAN

A member of the patient's treatment team, the protocol investigator, or research team at Memorial Sloan Kettering Cancer Center will identify potential research participants. If the investigator is a part of the treatment team, s/he will screen the patient as to eligibility, and will discuss the study and the possibility of enrollment in the research study with the patient. The preliminary screen of eligibility will be confirmation for the diagnosis of the following:

- **Patients with metastatic EGFR mutant lung cancers**

Potential subjects that meet these basic criteria will be referred by their treatment physician to the investigator, co-investigators, or research staff of the study. Minority and women are well represented in the thoracic oncology clinics, and we expect that they will be well represented in the trial accrual. The principal investigator**, Dr. Helena Yu**, will be available to all patients for further questions and information through a contact number which will be provided on the consent form itself.

During the initial conversation between the investigator/research staff and the patient, the patient may be asked to provide certain health information that is necessary to the recruitment and enrollment process. The investigator/research staff may also review portions of their medical records at MSKCC in order to further assess eligibility. In particular, pathology reports including IMPACT results/IHC results will be reviewed to see if the patient meets eligibility for the study. They will use the information provided by the patient and/or medical record to confirm that the patient is eligible and to contact the patient regarding study enrollment. If the patient turns out to be ineligible for the research study, the research staff will destroy all information collected on the patients during the initial conversation and medical records review.

All recruited patients will be under the care of attending medical oncologists of the MSKCC Thoracic Oncology Service. There will be no direct advertising for this study and participants will not be reimbursed for participation. Patients will be accrued to this study without regard for gender or minority status. The study will be available to the public and the details of the inclusion criteria, exclusion criteria and study design will be posted at www.clinicaltrials.gov.

8.0 PRETREATMENT EVALUATION

The following tests must be completed within 28 days of started treatment on study unless otherwise noted.

- Documented presence of the EGFR mutation within the patient's tumor (no time window)
- Medical history
- Baseline tumor assessment with CT scan of the chest/abdomen/pelvis is preferred, or other comparable radiologic study (PET scan) can be utilized if adequate assessment of target lesions can be performed.
- Baseline CNS assessment with contrast-enhanced MRI brain (or CT if MRI is contraindicated). This can be within 12 weeks of study start.
- Physical examination, vital signs (pulse, blood pressure, temperature, respiratory rate) as well as weight.
- 12-lead electrocardiogram (ECG) (within 12 weeks of study start)
- Performance status by KPS

- Serum or urine pregnancy test (for premenopausal women with child-bearing potential) within 7 days of treatment start
- Complete blood count with differential
- Chemistry evaluation including glucose, blood urea nitrogen, creatinine, sodium, potassium, chloride, bicarbonate, calcium, total protein, albumin, serum bilirubin, alkaline phosphatase, ALT, AST
- cfDNA plasma blood test

9.0 TREATMENT/INTERVENTION PLAN

Dacomitinib and osimertinib treatment

Phase 1: The study will assess whether combination dacomitinib and osimertinib is safe and tolerable in patients with metastatic EGFR-mutant lung cancers. There are four pre-planned dose-levels with escalating does of osimertinib and dacomitinib.

Table 9-1: Modeling derived dose levels:

Dose level	Dacomitinib	Osimertinib
Dose level 1	Dacomitinib 15mg daily	Osimertinib 40mg daily
Dose level 2	Dacomitinib 15mg daily	Osimertinib 40mg BID
Dose level 3	Dacomitinib 30mg daily	Osimertinib 40mg BID

Both dacomitinib and osimertinib can be taken with or without food. They can be taken together or separately. There does not need to be time in between dosing of each drug. The time of dosing of each drug needs to be noted in the pill diary provided. If the patient is scheduled to come to clinic (i.e. CxD1), the patient should not take the study drugs until after they are evaluated in clinic.

Patients will begin on combination dacomitinib and osimertinib at the prescribed doses. A cycle will be 28 days in duration. The study will use a standard 3+3 dose escalation design. Three patients will need to be enrolled at each dose level and assessed for DLT for 1 full cycle (28 days for cycle 1) before dose escalation decision is made. Please see section 14.0 for dose escalation rules. 6 patients will need to be treated at a dose level for it to be determined the maximum tolerated dose (MTD). Once the MTD is established, there will be an expansion cohort of an additional 10 patients treated at the recommended phase 2 dose, likely the MTD to further assess safety and preliminary efficacy. Over time, if there are intolerable grade 1-2 toxicities that emerge necessitating dose reduction in ≥25% of subjects, we may consider a dose lower than the MTD as the recommended phase 2 dose.

Toxicity will be graded according to NCI CTCAE version 5. Response to therapy will be assessed with serial CT imaging every 2 cycles with CT scan of the chest/abdomen/pelvis with response evaluated by RECIST 1.1. Dose reductions after the initial DLT period can be made and are detailed in Section 11.0. Intra-patient dose-escalation will be permitted to dose levels that have already been evaluated and deemed safe as part of the 3+3 design. If a patient remains on a dose level for at least 2 cycles with ≤grade 1 toxicities only and the patient and their provider agree, they can escalate treatment to the next dose level (i.e. if someone started on dose level 1, was stable without > grade 1 toxicities, they could increase to dose level 2 at cycle 3 or later).

Correlative Studies

IMPACT will be done on archival tissue obtained before treatment on protocol. We will note if any molecular findings (C797S, EGFR amplification, HER2 amplification, etc) are associated with response to combination therapy. In addition, at screening, every 2 cycles and at disease progression, we will obtain a plasma sample for molecular testing. Allele frequency of specific EGFR mutations (sensitizing EGFR mutation, T790M, C797S) will be quantified and followed over time. A rebiopsy at the time of clinical progression on EGFR TKI will not be mandated per protocol (optional) but is typically done as per standard of care. We will collect molecular data from this repeat biopsy and attempt to identify potential mechanisms of resistance to combination dacomitinib/osimertinib therapy.

10.0 EVALUATION DURING TREATMENT/INTERVENTION

The table below outlines the schedule of assessments applied to patients. All assessmentshave a window of +/- 7 days unless otherwise noted. If the subject is unable to have a study assessment taken within the defined time window due to an event outside of his or her control (e.g. clinic closure, personal emergency, inclement weather, vacation) the assessment should be performed as close as possible to the required schedule. Tumor assessments have a window of +/- 2 weeks. If an appropriate imaging study is done for an unrelated reason, it can be used for disease assessment if it falls within the appropriate time frame. Subjects will return to the study site within 30 days after their last dose of study drugs to complete end of study assessments outlined below.

Table 10-1: Study Evaluations

**Study Assessments**	**Screening**	**Cycle 1**		**Cycle 2+**	**Off Treatment**	**Survival Follow-up**
Day	Within 4 weeks except as noted	C1D1	C1D15		w/in 30 days	
Informed Consent	X					
Medical History	X	X	X	X	X	
Physical Exam with KPS[10]	X	X	X	X	X	
Vital Signs	X	X	X	X	X	
Adverse Events		X	X	X	X	
Systemic tumor Assessment[1]	X			X	X	
CNS tumor assessment[1]	X (w/in 12 weeks)			X		
12-lead EKG	X (w/in 12 weeks)					
Echocardiogram	X (w/in 12 weeks)			X[8]		
CBC[2]	X	X		X	X	
Chemistries[3]	X	X		X	X	
Pregnancy Test	X[9]					
IMPACT testing[4]	X (no time limit)				X	

Tumor biopsy[5]	X (no time limit)				X (optional)	
Plasma cfDNA collection[6]	X			X	X	
Drug Administration[7]		X		X		
Annual chart review						X

1. CT chest/abdomen/pelvis with or without contrast (preferred and will be used for all subsequent scans after screening). CT will be done at screening, every 2 cycles (+/- 2 weeks) and at progression, or when patient comes off study. An MRI of the brain, or a comparable study, will be done every 4 cycles (+/- 2 weeks) for patients with no brain metastases. For patients with brain metastases, MRI will be done at screening, every 2 cycles (+/- 2 weeks) and at progression, or when patient comes off study.
2. CBC- complete blood count with differential
3. Chemistries- to include glucose, blood urea nitrogen, creatinine, sodium, potassium, chloride, bicarbonate, calcium, total protein, albumin, serum bilirubin, alkaline phosphatase, ALT, AST)
4. IMPACT molecular profiling will be done on pre-treatment archived tissue if available. If a post-progression biopsy is performed as standard of care, IMPACT molecular results will be obtained. A post-progression biopsy is not mandatory or included in the protocol.
5. A tumor biopsy must have occurred prior to study treatment with available archived tissue for IMPACT testing. A repeat biopsy after progression on EGFR TKI is standard of care, and patients after progression on this treatment regimen will be sent for a repeat biopsy. The post-treatment biopsy is optional.
6. Plasma cfDNA collection will occur during screening, at followup1 scan and at end of treatment/Progression.
7. Dacomitinib and osimertinib are dosed orally daily or twice daily per study schedule. Pills will be dispensed on the first day of every cycle.
8. Echocardiogram will be done every 3 cycles (12 weeks +/- 2 weeks)
9. Serum or urine pregnancy test for pre-menopausal woman with child-bearing potential must be assessed within 7 days of study drug initiation.
10. KPS – Karnofsky Performance Status

11.0 TOXICITIES/SIDE EFFECTS

Adverse events occurring in patient may be attributable to dacomitinib, osimertinib or the combination. Dose modifications or discontinutations should be based on the nature, severity, and attributions of the AEs and are at the discretion of the treating physician and/or principal investigator. General guidelines are provided below. For safety and adverse event reporting, see **Section 17.0.**

11.1 Dacomitinib Related Toxicities:

Toxicities with dacomitinib that are likely (>20%) include:

- Rash
- Diarrhea
- Nail infections and inflammation around the nail folds
- Mouth sores
- Decreased appetite
- Dry or cracked skin
- Decreased weight
- Nausea

Toxicities with dacomitinib that are less likely (<20%) include:

- Itchy skin
- Cough
- Elevation in liver function blood tests
- Inflammation of the eye, dry eyes
- Redness/swelling/peeling of the palms and the soles
- Cough
- Shortness of breath

- Decreased energy
- Constipation
- Hair loss
- Pain in legs and arms
- Nosebleeds
- Abnormal hair growth

Side effects of dacomitinib that are rare, but serious include:

- Low blood levels of potassium
- Infections like pneumonia
- Allergic skin reaction that causes a flat, itchy red rash with bumps and peeling of the skin
- Inflammation of the lungs causing shortness of breath

11.2 Dose modifications of dacomitinib

Dose modifications of dacomitinib are allowed and are at the discretion of the principal investigator and treating physician. If a patient experiences a CTCAE grade 3 or higher and/or unacceptable toxicity (any grade), where the Investigator considers the AE to be associated with one drug not the other, dosing of the culprit drug will be interrupted, non-culprit drug continued and supportive therapy administered as required in accordance with local practice/guidelines. If an AE is not clearly attributable to one drug, both drugs will be held until the AE resolves as specified below. If patients have intolerable grade 1/2 toxicities, dose modifications can be made at the discretion of the treating physician. The principal investigator is available should there be any questions regarding dose modifications.

Guidelines for dose reductions and interruptions are as follows:

Table 11-1: Dacomitinib Dose modifications (Cycle ≥2 only)

Dose at time of toxicity	Dose reduction
Dacomitinib 15mg daily	• Dacomitinib 15mg 3x/week<br>• Consider discontinuation of dacomitinib
Dacomitinib 30mg daily	• Dacomitinib 15mg daily

<u>11.3 Osimertinib Related Toxicities:</u>

Toxicities with Osimertinib that are likely (>20%) include:

- Rash
- Diarrhea
- Nausea
- Decreased appetite
- Dry skin

Toxicities with Osimertinib that are less likely (<20%) include:

- Itchy skin
- Decreased energy
- Nail changes
- Mucositis
- Constipation
- Cough

- Vomiting
- Low red blood cell counts which can lead to fatigue
- Shortness of breath
- Upper respiratory tract infections
- Headache

Side effects of Osimertinib that are rare, but serious include:

- Pneumonitis (inflammation of the lung). Fatal events of inflammation of lungs have been reported in patients taking Osimertinib.

11.4 Dose modifications

11.4.1 Dose modifications of osimertinib

Dose modifications of osimertinib are allowed and are at the discretion of the principal investigator and treating physician. Guidelines for dose reductions and interruptions are as follows:

Table 11-2: Osimertinib Dose modifications (Cycle ≥2 only)

Dose at time of toxicity	Dose reduction levels
Osimertinib 40mg QD	• Osimertinib 40mg every other day<br>• Consider discontinuation of Osimertinib
Osimertinib 40mg BID	• Osimertinib 40mg QD
Osimertinib 80mg BID	• Osimertinib 40mg BID

For any toxicity, supportive medications should be utilized. For example, for skin reactions, mild to moderate strength steroids creams, either topical or systemic antibiotics should be started, as seen appropriate by the investigator upon assessment of the skin reaction. For nausea/vomiting, anti- emetic therapy should be utilized. For diarrhea, after alternative etiologies are ruled out, anti-diarrheal medications should be utilized.

If a patient experiences a CTCAE grade 3 or higher and/or unacceptable toxicity (any grade), where the Investigator considers the AE to be associated with one drug not the other, dosing of the culprit drug will be interrupted, non-culprit drug continued and supportive therapy administered as required in accordance with local practice/guidelines. If an AE is not clearly attributable to one drug, both drugs will be held until the AE resolves as specified below. If patients have intolerable grade 1/2 toxicities, dose modifications can be made at the discretion of the treating physician. The principal investigator is available should there be any questions regarding dose modifications.

If the toxicity resolves or reverts to ≤CTCAE grade 1 or tolerable grade 2, study drug may be restarted at the same dose (starting dose) or reduced dose using the dose reductions in the tables above. If restarting at the same dose level, patients should be closely monitored for 3 days following the restart of treatment. They will call should they have any issues and during that 3 day period a nurse will call them for reassessment. If within 3 days there is recurrence of same toxicity, a dose reduction should be considered at the Investigator’s discretion. For grade 4 treatment-related toxicities that occur during any cycle, a dose reduction will be mandated after drug dosing is interrupted. If a grade 3 or interolable grade 2 treatment-related toxicity occurs, a dose reduction may be instituted at the investigators discretion.

If the toxicity does not resolve to ≤CTCAE grade 1 or tolerable grade 2 after 3 weeks, then the culprit study drug should be permanently discontinued. If one drug is permanently discontinued,

it is the treating physician's discretion as to whether to continue the other drug as monotherapy. If both drugs are culprits and are discontinued, the subject would come off study for toxicity.

Patients who develop asymptomatic grade 3 or grade 4 lymphopenia without clinical correlate (eg. Opportunistic infection) may continue study treatment without interruption if approved by the treating physician.

Interstitial lung disease/pneumonitis:
If a new or worsening of pulmonary symptoms (e.g., dyspnea) or occurrence of a radiological abnormality suggestive of ILD is observed (any grade), an interruption in study drug dosing (both osimertinib and dacomitinib) is recommended. In the presence of confirmatory CT scans where other causes of respiratory symptoms have been excluded, a diagnosis of ILD should be considered and study drug permanently discontinued. In the absence of a diagnosis of ILD, study drug may be restarted following consultation with the Principal investigator and sponsor.

Table 11-3: QTC Prolongation

Toxicity grade	Action/Dose modification
1 or 2	No action needed.
3	Hold osimertinib. Regular ECGs (at least weekly) until resolution of QTcF <481 ms and then restart osimertinib at one reduced dose level. If toxicity does not resolve to QTcF<481 within 21 days, permanently discontinue osimertinib
4	Discontinue study drug permanently and seek cardiac evaluation.

Congestive Heart Failure:
LVEF will be assessed by echocardiogram during screening and every 12 weeks. If ejection fraction decreased by $\geq$10% from pretreatment values AND is less than 50%, hold osimertinib. For symptomatic congestive heart failure OR persistent, asymptomatic LV dysfunction that does not resolve within 4 weeks, permanently discontinue osimertinib. If LVEF improves to baseline within 4 weeks, consider restarting drug at one reduced dose level and/or discontinue permanently as per investigator's discretion.

Table 11-4: Dose modification chart

Toxicity	Grade 1	Grade 2	Grade 3	Grade 4
Non-hematologic, general (except for what is noted below)*	Continue at same dose level. Can dose reduce for intolerable grade 1/2 toxicities per investigator discretion.	Continue at same dose level. Can dose reduce for intolerable grade 1/2 toxicities per investigator discretion.	Withhold dose until toxicity is grade ≤ 1, then resume treatment at same dose level, or dose level -1, at the discretion of the investigator	Withhold dose until toxicity is grade ≤ 1, then resume treatment at same dose level, or dose level -1, at the discretion of the investigator
Diarrhea	Continue at same dose level. Initiate therapy with loperamide. Can dose reduce for intolerable grade 1/2 toxicities per investigator discretion.	Continue at same dose level. Initiate therapy with loperamide. Can dose reduce for intolerable grade 1/2 toxicities per investigator discretion.	Initiate therapy with loperamide. Withhold until toxicity is grade ≤ 2. Resume treatment at same dose level or dose level -1 at discretion of investigator.	Initiate therapy with loperamide. Withhold until toxicity is grade ≤ 2. Resume treatment at same dose level or dose level -1 at discretion of investigator.
Rash	Continue at same dose level. Supportive symptom management. Can dose reduce for intolerable grade 1/2 toxicities per	Continue at same dose level. Supportive symptom management. If rash persists or worsens over 14 days, reduce by 1 dose level. Can dose reduce for	Reduce by 1 dose level. Supportive symptom management and initiate supportive symptom management. If rash persists of worsens	Withhold until toxicity is grade ≤ 2. Initiate supportive symptom management. Discontinue permanently or restart at dose level -

	investigator discretion.	intolerable grade 1/2 toxicities per investigator discretion.	over 14 days, interrupt drug until resolution to ≤ grade 2, and resume dose level -1 or -2 at discretion of investigator.	2 at discretion of investigator.

11.4.2 Dose modifications of dacomitinib

Dose modifications of dacomitinib are allowed and are at the discretion of the principal investigator and treating physician. Guidelines for dose reductions and interruptions are as follows:

Table 11-5: Dacomitinib Dose modifications (Cycle ≥2 only)

Dose at time of toxicity	Dose reduction levels
Dacomitinib 15mg daily	• Dacomitinib 15mg 3x/week<br>• Consider Dacomitinib discontinuation
Dacomitinib 30mg daily	• Dacomitinib 15mg daily

For any toxicity, supportive medications should be utilized. For example, for skin reactions, mild to moderate strength steroids creams, either topical or systemic antibiotics should be started, as seen appropriate by the investigator upon assessment of the skin reaction. For nausea/vomiting, anti- emetic therapy should be utilized. For diarrhea, after alternative etiologies are ruled out, anti-diarrheal medications should be utilized.

If a patient experiences a CTCAE grade 3 or higher and/or unacceptable toxicity (any grade), where the Investigator considers the AE to be associated with one drug not the other, dosing of the culprit drug will be interrupted, non-culprit drug continued and supportive therapy administered as required in accordance with local practice/guidelines. If an AE is not clearly attributable to one drug, both drugs will be held until the AE resolves as specified below. If patients have intolerable grade 1/2 toxicities, dose modifications can be made at the discretion of the treating physician. The principal investigator is available should there be any questions regarding dose modifications.

If the toxicity resolves or reverts to ≤CTCAE grade 1 or tolerable grade 2, study drug may be restarted at the same dose (starting dose) or reduced dose using the dose reductions in the tables above. If restarting at the same dose level, patients should be closely monitored for 3 days following the restart of treatment. They will call should they have any issues and during that 3 day period a nurse will call them for reassessment. If within 3 days there is recurrence of same toxicity, a dose reduction should be considered at the Investigator's discretion. For grade 4 treatment-related toxicities that occur during any cycle, a dose reduction will be mandated after drug dosing is interrupted. If a grade 3 or interolable grade 2 treatment-related toxicity occurs, a dose reduction may be instituted at the investigators discretion.

If the toxicity does not resolve to ≤CTCAE grade 1 or tolerable grade 2 after 3 weeks, then the culprit study drug should be permanently discontinued. If one drug is permanently discontinued, it is the treating physician's discretion as to whether to continue the other drug as monotherapy. If both drugs are culprits and are discontinued, the subject would come off study for toxicity.

Table 11-6: Dose modification chart

Toxicity	Grade 1	Grade 2	Grade 3	Grade 4
Non-hematologic, general (except for what is noted below)*	Continue at same dose level. Can dose reduce for intolerable grade 1/2 toxicities per investigator discretion.	Continue at same dose level. Can dose reduce for intolerable grade 1/2 toxicities per investigator discretion.	Withhold dose until toxicity is grade ≤ 1, then resume treatment at same dose level, or dose level -1, at the	Withhold dose until toxicity is grade ≤ 1, then resume treatment at same dose level, or dose level -1, at the

			discretion of the investigator	discretion of the investigator
Diarrhea	Continue at same dose level. Initiate therapy with loperamide. Can dose reduce for intolerable grade 1/2 toxicities per investigator discretion.	Continue at same dose level. Initiate therapy with loperamide. Can dose reduce for intolerable grade 1/2 toxicities per investigator discretion.	Initiate therapy with loperamide. Withhold until toxicity is grade ≤ 2. Resume treatment at same dose level or dose level -1 at discretion of investigator.	Initiate therapy with loperamide. Withhold until toxicity is grade ≤ 2. Resume treatment at same dose level or dose level -1 at discretion of investigator.
Rash	Continue at same dose level. Supportive symptom management. Can dose reduce for intolerable grade 1/2 toxicities per investigator discretion.	Continue at same dose level. Supportive symptom management. If rash persists or worsens over 14 days, reduce by 1 dose level. Can dose reduce for intolerable grade 1/2 toxicities per investigator discretion.	Reduce by 1 dose level. Supportive symptom management and initiate supportive symptom management. If rash persists of worsens over 14 days, interrupt drug until resolution to ≤ grade 2, and resume dose level -1 or -2 at discretion of investigator.	Withhold until toxicity is grade ≤ 2. Initiate supportive symptom management. Discontinue permanently or restart at dose level -2 at discretion of investigator.

<u>11.5 Dose Limiting Toxicities:</u>

Dose Limiting Toxicities are adverse events (graded according to CTCAE v5) experienced in the first cycle of treatment (i.e. 4 weeks) that include any of the following:

- Death related to the investigational regimen
- Hematologic toxicities as follows:
  - Grade = 4 neutropenia lasting > 5 days
  - Grade = 4 thrombocytopenia (<25,000/mm3)
  - Grade ≥ 3 thrombocytopenia with evidence of clinically significant bleeding
  - Grade = 4 anemia
- Non-hematologic toxicities as follows:
  - Grade ≥ 3 AST, ALT, alkaline phosphatase or total bilirubin that is confirmed on repeat labs ≥ 72 hours after initial labs obtained
  - Grade ≥ 3 diarrhea, nausea, vomiting that lasts > 72 yrs despite optimal maximal supportive care
  - Grade 4 diarrhea (life-threatening consequences; urgent intervention indicated) regardless of duration
  - Grade 4 vomiting (life-threatening consequences) regardless of duration
  - Any other non-hematologic grade ≥ 3 major organ toxicity
  - Any adverse event (regardless of grade) requiring a dose delay of ≥ 2 weeks
  - Any adverse even requiring more than 2 dose reductions

In the event that 2 or more patients in a given treatment cohort experience a DLT, that cohort will be closed.

12.0 CRITERIA FOR THERAPEUTIC RESPONSE/OUTCOME ASSESSMENT

Best overall response rate (confirmed partial and complete responses) will be assessed as part of this study. All responses must be confirmed on subsequent scan to be considered a

confirmed response.Tumor response will be assessed using RECIST 1.1. The same method of assessment (CT scan or MRI) and the same technique (i.e. with or without contrast) should try to be used to characterize each identified and reported lesion at baseline, on study and at end of treatment. Designated radiologists at MSKCC (named on the study face sheet) will interpret the study CTs according to RECIST 1.1 criteria in addition to the principal investigator.

For most patients, a CT chest/abdomen/pelvis will be performed to demonstrate all known areas of measurable disease. The baseline study will occur no more than 4 weeks prior to first study drug administration. A baseline PET/CT can be used for screening if target lesions can be adequately measured. All patients must have at least one measurable disease lesion by CT. MRI brains (+/- spine) will be required as well to assess CNS involvement.

Target and non target lesions

All measurable lesions, up to a maximum of 5 lesions total, representative of all involved organs should be identified as target lesions and recorded and measured at baseline. Target lesions should be selected on the basis of their size, should be representative of all involved organs, and should lend themselves to reproducible repeat measurements. All measurements should be recorded in millimeters. All other lesions (or sites of disease) should be identified as non-target lesions and should also be recorded at baseline as well. Measuresments of nontarget lesions are not required but should be followed as “present”, “absent” or in rare cases “unequivocal progression”.

Tumor response evaluation

Definitions of response in target and non-target lesions are described in Table 12-1 and 12-2 below. Table 12-3 provides overall responses for all possible combinations of tumor responses in target and nontarget lesions.

**Table 12-1: Evaluation of target lesions**	
Complete Response (CR):	Disappearance of all target lesions
Partial response (PR)	At least a 30% decrease in the sum of the diameters of the target lesions
Progressive disease (PD):	At least a 20% increase in the sum of the diameter of the target lesions or the appearance of one or more new lesions
Stable disease (SD):	Neither sufficient shrinkage to qualify for PR nor sufficient increase to qualify for PD

**Table 12-2: Evaluation of non-target lesions**	
Complete Response (CR):	Disappearance of all non-target lesions
Incomplete response/Stable disease (SD):	Persistence of one or more non-target lesions
Progressive disease (PD):	Appearance of one or more new lesion and/or unequivocal progression of existing non-target lesion

Table 12-3: Combinations of responses

**Target lesions**	**Nontarget lesions**	**New lesions**	**Overall response**
CR	CR	No	CR
CR	Incomplete/SD	No	PR
PR	Non-PD	No	PR
SD	Non-PD	No	SD
PD	Any	Yes or No	PD
Any	PD	Yes or No	PD
Any	Any	Yes	PD

Confirmation of scans

Verification of response: Initial observations of responses will be confirmed by repeat scans which should be performed no earlier than 4 weeks after the original observation to ensure that responses identified are not the result of measurement error. After an initial PR or CR is noted, the subsequent protocol-specified tumor assessment may serve as the confirmation.

Verification of progression: profression of disease should be verified in cases where progression is equivocal. If repat scans confirm PD, then progression should be declared using the date of the initial scan.

Evaluation of best overall response

The best overall response is the best response recorded from the start of treatment until disease progression, as defined in Table 12-3.

Special consideration of new lesions:

The appearance of new malignant lesions denotes disease progression typically if the longest diameter is least 10mm. New lesions may be defined by any modality (CT, PET, MRI, clinical exam). The finding of a new lesion should be unequivocal (i.e. not attributable to differences in scanning technique, change in imaging modality or findings that may represent other processess (infection, inflammation). This is particularly importanty when the patient's baseline lesions continue to show partial or complete response. If a new lesion is equivocal, for example because of small size or indeterminate etiology, therapy may be continued and follow-up examination will clarify if it represents new disease.

Other definitions

Evaluable for dose-escalation design decisions: Patients that successfully complete Cycle 1 of treatment will be considered evaluable in the dose-escalation portion of the study. Patients that discontinue treatment for reasons unrelated to a DLT will be deemed inevaluable.

Evaluable for toxicity: All patients who receive at least one dose of study therapy will be evaluable for toxicity.

Evaluable for efficacy: All patients will be included in the analysis of efficacy.

Progression-free survival (PFS) is defined as the duration of time from first treatment to time of progression (in the CNS or systemically) or death, whichever occurs first.

Intracranial progression-free survival (PFS) is defined as the duration of time from first treatment to time of progression (in the CNS) or death, whichever occurs first.

Overall survival (OS) is defined as the duration of time from first treatment to time of death.

13.0 CRITERIA FOR REMOVAL FROM STUDY

Patients may withdraw from the study at any time. Other reasons for study discontinuation include but are not limited to:

- Change in patient eligibility
- Non-compliance with the defined treatment plan
- Protocol deviation
- Investigator's decision based on patient's best interest
- Withdrawal of consent
- Lost to follow up
- Death
- Progression of disease (defined by RECIST 1.1)

14.0 BIOSTATISTICS

Phase 1 Escalation Cohort
Primary objective: to determine maximum tolerated dose of the combination of dacomitinib and osimertinib in patients with *EGFR* mutated advanced non-small cell lung cancer (NSCLC).

Primary endpoint: identify any dose limiting toxicities, and establish maximum tolerated dose for the combination. The MTD will be defined as the highest dose at which not more than 1/6 of the patients experience dose limiting toxicity (DLT). DLT is defined as any of the toxicity events described below that occurs within cycle 1 of treatment with the combination of dacomitinib and osimertinib.

Secondary Objectives: 1) measure best overall response, 2) measure progression-free survival (PFS) 3) measure overall survival (OS) 4) determine the frequency of EGFR T790M and C797S emergence on this combination regimen.

The NCI Common Terminology Criteria for Adverse Events Version 5 (NCI-CTCAE) will be used to grade toxicities during the trial. Dose-limiting toxicities (DLT's) are defined as any of the following events occurring during the first cycle of treatment (i.e. 4 weeks):

- Death related to the investigational regimen
- Hematologic toxicities as follows:
  - Grade = 4 neutropenia lasting > 5 days
  - Grade = 4 thrombocytopenia (<25,000/mm3)
  - Grade ≥ 3 thrombocytopenia with evidence of clinically significant bleeding
  - Grade = 4 anemia
- Non-hematologic toxicities as follows:
  - Grade ≥ 3 AST, ALT, alkaline phosphatase or total bilirubin that is confirmed on repeat labs ≥ 72 hours after initial labs obtained
  - Grade ≥ 3 diarrhea, nausea, vomiting that lasts > 72 yrs despite optimal maximal supportive care
  - Grade 4 diarrhea (life-threatening consequences; urgent intervention indicated) regardless of duration
  - Grade 4 vomiting (life-threatening consequences) regardless of duration
  - Any other non-hematologic grade ≥ 3 major organ toxicity
  - Any adverse event (regardless of grade) requiring a dose delay of ≥ 2 weeks
  - Any adverse even requiring more than 2 dose reductions

If a DLT begins in the first cycle (4 weeks) that by definition requires a minimum duration of time to be declared, that toxicity must be followed out past cycle 1 to confirm it does or does not meet DLT criteria. The patients in a cohort must complete the first 4 weeks of study therapy and any additional time needed to determine whether DLT criteria is met prior to enrollment of subsequent cohorts.

If no DLT is seen in the first 3 patients at a dose level (i.e. dose level 1), the following 3 patients will be enrolled at the next dose level (i.e. dose level 2). If 1 DLT is observed in the first cohort, the next 3 patient cohort will be enrolled at the same dose level to expand the cohort at that dose level to 6 patients. If no further DLTs are identified in the expanded cohort (i.e. 0-1 DLT for 6 patients at the same dose level), then the following 3 patient cohort will be enrolled at the next dose level. If 2 DLTs are observed in any observed 3 patient cohort, the next 3 patient cohort will be enrolled at the immediately previous lower dose level to expand the number of patients at that level to 6 patients and no further dose escalation will occur.

Table 14-1: Modeling derived dose levels

Dose level	Dacomitinib	Osimertinib
Dose level 1	Dacomitinib 15mg daily	Osimertinib 40mg daily
Dose level 2	Dacomitinib 15mg daily	Osimertinib 40mg BID
Dose level 3	Dacomitinib 30mg daily	Osimertinib 40mg BID

If 2 DLTs are observed in dose level 1, the protocol may be amended to consider alternative dose levels. The MTD will be defined as the highest dose where not more than 1 of 6 patients develops a DLT. Dose levels are outlined in table above. Dose escalation will proceed within each cohort according to the schema in Table below. Patients who do not complete 28 days of treatment for reasons other than experiencing a DLT will be replaced. For the purposes of dose-escalation decisions, only DLTs occurring during the first cycle (28 days) will be considered.

Table 14-2: Dose Escalation Schema

**# of pts with DLT at a given dose level**	**Escalation decision rule**
0 of 3 or 1 of 6	Enter next cohort (3 pts) at next dose level
1 of 3	Enter next cohort (3 pts) at same dose level<br>• If 0 of 3 experience DLT, proceed to next dose level<br>• If 1 or more experience DLT, dose escalation stopped. 3 additional pts will be entered at next lowest dose level if only 3 patients were treated previously at that dose level
≥2 of 3 or ≥2 of 6	Dose escalation stopped. 3 additional pts will be entered at next lowest dose level if only 3 patients were treated previously at that dose level

Table 14-3:The Probability of Escalation Given Different True Rates of Dose-Limiting Toxicity

**Toxicity rate**	0.10	0.20	0.30	0.40	0.50
**Probability of escalation**	91%	71%	49%	31%	17%

Response to therapy will be assessed by interval imaging approximately every 8 weeks (+/- 2 week) with high resolution CT scan (or other comparable radiologic study) with response evaluated per RECIST 1.1. Responses (PR/CR) will be summarized by dose level; for patients treated at MTD, the corresponding response rate will be reported along with exact 95%CI. Progression-free survival and overall survival will be estimated using Kaplan-Meier method starting from time of the first dose of study therapy.

Phase 1 Expansion cohort

Primary objective: to further establish the toxicity profile of the combination of combination dacomitinib and osimertinib.

Secondary objective: to obtain preliminary efficacy data by measuring the objective response rate (sum of complete responses and partial responses according to RECIST 1.1), progression free survival and overall survival.

After identification of the MTD, 6 patients will have been treated at the MTD. An additional 10 patients will be enrolled at the recommended phase 2 dose, likely the MTD, for further evaluation of safety and preliminary evaluation of efficacy. If 3 or more DLTs are observed among the first 6 expansion patients or 4 or more DLTs are observed among the 10 expansion patients the MTD will

be deemed as too high. Further evaluation at lower doses will be needed to determine the recommended dose for phase 2.

Table 14-4:The Operating Characteristics of This Stopping Rule

True tox rate	0.2	0.26	0.32	0.38	0.44	0.50
Prob of stopping for toxicity	0.154	0.291	0.448	0.605	0.742	0.848

Secondary objective:

Objective response rate (CR+PR) will be calculated along with exact 95% CI for the 16 patients treated at MTD. PFS and OS will be estimated by Kaplan-Meier method for the 16 patients. The time origin for PFS and OS is treatment start date. The frequency of EGFR T790M and C797S emergence on this combination regimen will be summarized by descriptive statistics.

The minimum number of patients to identify the MTD would be 8, the maximum being 24. When including the 10 person expansion cohort, the total number of patients enrolled in the phase 1 study will be 2-36. Given an expected accrual rate of 2-3 patients per month, accrual will be completed in about 1.5 years.

Correlative Studies:
Objectives: 1) identify any pre-treatment biomarkers that predict response to combination osimertinib/dacomitinib in this setting 2) identify molecular biomarkers within plasma (sensitizing EGFR mutation, EGFR C797S, EGFR T790M), quantify and follow serially over time. 3) determine the mechanisms of resistance to osimertinib/dacomitinib treatment

Correlatives:
For the correlative studies, the analysis is primarily exploratory and hypothesis generating. Correlative studies will be performed on all patients in the phase 1 and expansion cohorts including descriptive summaries of mutation testing. IMPACT analyses which will be limited to patients with tumor samples available. Tissue will only be available after protocol treatment from subjects who agree to a standard of care post-progression biopsy. While all the genes in the IMPACT panel will be assessed, only those with at least 10% prevalence in this trial will be statistically analyzed.

Frequency of EGFR alterations in cfDNA in plasma will be determined pre-treatment, at first followup scan and post treatment/at progression in those patients who progress.The frequency of EGFR mutations in both tumor tissue and plasma will be calculated along with the exact 95% confidence interval. The pre- treatment frequency of concurrent alterations identified by the IMPACT panel will be univariately correlated with response using Fisher’s exact test and with PFS and OS using logrank test.

To evaluate the mechanism of resistance to osimertinib and dacomitinib, changes in mutation frequency over time (before treatment, on treatment and at progression) will be reported descriptively. We will also estimate the frequency of EGFR T790M/EGFR C797S at progression and compare it to that of patients treated with standard of care by binomial test.

15.0 RESEARCH PARTICIPANT REGISTRATION AND RANDOMIZATION PROCEDURES

15.1 Research Participant Registration

Confirm eligibility as defined in the section entitled Inclusion/Exclusion Criteria. Obtain informed consent, by following procedures defined in section entitled Informed Consent Procedures. During the registration process registering individuals will be required to

complete a protocol specific Eligibility Checklist. The individual signing the Eligibility Checklist is confirming whether or not the participant is eligible to enroll in the study. Study staff are responsible for ensuring that all institutional requirements necessary to enroll a participant to the study have been completed. See related Clinical Research Policy and Procedure #401 (Protocol Participant Registration).

16.0 DATA MANAGEMENT ISSUES

A Clinical Research Coordinator (CRC) will be assigned to the study. The responsibilities of the CRC include project compliance, data collection, abstraction and entry, data reporting, regulatory monitoring, problem resolution and prioritization, and coordinate the activities of the protocol study team. The data collected for this study will be entered into MSK Medidata. Source documentation will be available to support the computerized patient record. The principal investigator will maintain ultimate responsibility for the clinical trial.

16.1 Quality Assurance

Weekly registration reports will be generated to monitor patient accruals and completeness of registration data. Routine data quality reports will be generated to assess missing data and inconsistencies. Accrual rates and extent and accuracy of evaluations and follow-up will be monitored periodically throughout the study period and potential problems will be brought to the attention of the study team for discussion and action. Random-sample data quality and protocol compliance audits will be conducted by the study team, at a minimum of two times per year, more frequently if indicated.

16.2 Data and Safety Monitoring

The Data and Safety Monitoring (DSM) Plans at Memorial Sloan-Kettering Cancer Center were approved by the National Cancer Institute in September 2001. The plans address the new policies set forth by the NCI in the document entitled "Policy of the National Cancer Institute for Data and Safety Monitoring of Clinical Trials" which can be found at: http://www.cancer.gov/clinicaltrials/patientsafety/dsm-guidelines/page1

The DSM Plans at MSKCC were established and are monitored by the Office of Clinical Research. The MSKCC Data and Safety Monitoring Plans can be found on the MSKCC Intranet at:http://smskpsps9/dept/ocr/OCR%20Website%20Documents/Clinical%20Research%20Quality%20Assurance%20(CRQA)/MSKCC%20Data%20and%20Safety%20Monitoring%20Plan.pdf

There are several different mechanisms by which clinical trials are monitored for data safety and quality. There are institutional processes in place for quality assurance (e.g., protocol monitoring, compliance and data verification audits, therapeutic response, and staff education on clinical research QA) and departmental procedures for quality control, plus there are two institutional committees that are responsible for monitoring the activities of our clinical trials programs. The Data and Safety Monitoring Committee (DSMC) reports to the Center's Research Council and Institutional Review Board.

During the protocol development and review process, each protocol will be assessed for its level or risk and degree of monitoring required. Every type of protocol (e.g., NIH sponsored, in-house sponsored, industry sponsored, NCI cooperative group, etc.) will be addressed and the monitoring procedures will be established at the time of protocol activation

17.0 PROTECTION OF HUMAN SUBJECTS

Prior to the enrollment of each patient, the risks, benefits and objectives of the study will be reviewed with the participant, including a discussion of the possible toxicities and side effects. Alternative, non-protocol, treatment options will be discussed with the patient. It will be reviewed that participation in this clinical trial is voluntary and that the patient may withdraw consent at any time. The study is designed with careful safety monitoring for toxicity including physician visits. Specific guidelines for symptom management are in place to protect the study participant.

Human Subjects Involvement and Characteristics: All patients at MSKCC who meet the inclusion criteria will be eligible. Up to 34 patients will be enrolled on study. Patients eligible will be 18 years of age or older with a KPS of 70% or greater. Both men and women and members of all ethnic groups are eligible for this trial. Pregnant and breast-feeding women are excluded from this study. This protocol does not include children because the number of children is expected to be limited for the patient population expected to be accrued onto this study. Also, the majority of children are already accessed by a nationwide pediatric cancer research network. This statement is based on exclusion 4b of the NIH Policy and Guidelines on the Inclusion of Children as Participants in Research Involving Human Subjects.

Consent process: All patients at MSKCC who meet the inclusion criteria will be eligible. Participation in the trial is voluntary. All patients will be required to sign a statement of informed consent, which must conform to IRB guidelines. The informed consent procedure is described in Section 18.0.

Possible Toxicities/Side-Effects: There are risks associated with treatment as described in Section 11.0; however, patients screened for enrollment will be deemed appropriate for treatment independent of this study.

Benefits: An alternative to study treatment would be osimertinib or other EGFR TKI single agent treatment. Both dacomitinib and osimertinib are established to be safe and efficacious in patients at typical doses in this treatment setting. It is not known whether combination treatment would be beneficial for patients. Patients who progress on the study treatment can still receive standard, second-line chemotherapy and/or can participate in an alternative clinical trial.

Costs: Patients will be charged for physician visits, routine laboratory tests and radiologic studies required for monitoring their condition. Pfizer Oncology will provide dacomitinib for study use. Osimertinib will be obtained commercially through patient insurance or other funds. Patients will be allowed to take 80mg Osi once a day for dose level 3, if insurance doesn't cover 40mg twice a day.The plasma testing and the correlative molecular analyses will be covered by research funds.

Alternatives: The alternative to this trial would be treatment with single agent treatment with FDA approved EGFR TKI (osimertinib, erlotinib, gefitinib, afatinib) or participation in another clinical trial.

Confidentiality: Every effort will be made to maintain patient confidentiality. Research and hospital records are confidential. Patients' names and any other identifying information will not be used in reports or publications resulting from this study. Other authorized agencies and appropriate internal personnel (e.g. qualified monitors from MSKCC) and external personnel, the FDA, and/or other governmental agencies) may review patient records as required.

Patient safety: Patients are monitored by physicians and oncology nurses who are very familiar with clinical trials. In the case of an adverse reaction, immediate medical attention is available. In the evenings and weekends, we have a 24-hour urgent care facility for outpatients. The PI or co-PI will also be available at all times to organize any necessary intervention.

Monitoring of data to ensure safety: This study is to be monitored by the institutional IRB. This incorporates an independent data and safety monitoring committee established by arrangement with the National Cancer Institute. The analysis of safety will include all patients. Adverse events, including all toxic effects of treatment, will be tabulated individually, and summarized by severity and causality.

17.1 Privacy

MSK's Privacy Office may allow the use and disclosure of protected health information pursuant to a completed and signed Research Authorization form. The use and disclosure of protected health information will be limited to the individuals described in the Research Authorization form. A Research Authorization form must be completed by the Principal Investigator and approved by the IRB and Privacy Board (IRB/PB).

The consent indicates that individualized de identified information collected for the purposes of this study may be shared with other qualified researchers. Only researchers who have received approval from MSK will be allowed to access this information which will not include protected health information, such as the participant's name, except for dates. It is also stated in the Research Authorization that their research data may be shared with other qualified researchers.

17.2 Serious Adverse Event (SAE) Reporting

An adverse event is considered serious if it results in ANY of the following outcomes:

- Death
- A life-threatening adverse event
- An adverse event that results in inpatient hospitalization or prolongation of existing hospitalization
- A persistent or significant incapacity or substantial disruption of the ability to conduct normal life functions
- A congenital anomaly/birth defect
- Important Medical Events (IME) that may not result in death, be life threatening, or require hospitalization may be considered serious when, based upon medical judgment, they may jeopardize the patient or participant and may require medical or surgical intervention to prevent one of the outcomes listed in this definition

Note: Hospital admission for a planned procedure/disease treatment is not considered an SAE.

SAE reporting is required as soon as the participant starts investigational treatment/intervention. SAE reporting is required for 30-days after the participant's last investigational

treatment/intervention. Any event that occur after the 30-day period that is unexpected and at least possibly related to protocol treatment must be reported.

Please note: Any SAE that occurs prior to the start of investigational treatment/intervention and is related to a screening test or procedure (i.e., a screening biopsy) must be reported.

All SAEs must be submitted in PIMS. If an SAE requires submission to the HRPP office per IRB SOP RR-408 'Reporting of Serious Adverse Events', the SAE report must be submitted within 5 calendar days of the event. All other SAEs must be submitted within 30 calendar days of the event.

The report should contain the following information:
- The date the adverse event occurred
- The adverse event
- The grade of the event
- Relationship of the adverse event to the treatment(s)
- If the AE was expected
- Detailed text that includes the following
    - o An explanation of how the AE was handled
    - o A description of the participant's condition
    - o Indication if the participant remains on the study
- If an amendment will need to be made to the protocol and/or consent form
- If the SAE is an Unanticipated Problem

For IND/IDE protocols:

The SAE report should be completed as per above instructions. If appropriate, the report will be forwarded to the FDA by the IND Office

18.0 INFORMED CONSENT PROCEDURES

Before protocol-specified procedures are carried out, consenting professionals will explain full details of the protocol and study procedures as well as the risks involved to participants prior to their inclusion in the study. Participants will also be informed that they are free to withdraw from the study at any time. All participants must sign an IRB/PB-approved consent form indicating their consent to participate. This consent form meets the requirements of the Code of Federal Regulations and the Institutional Review Board/Privacy Board of this Center. The consent form will include the following:

1. The nature and objectives, potential risks and benefits of the intended study.
2. The length of study and the likely follow-up required.
3. Alternatives to the proposed study. (This will include available standard and investigational therapies. In addition, patients will be offered an option of supportive care for therapeutic studies.)
4. The name of the investigator(s) responsible for the protocol.
5. The right of the participant to accept or refuse study interventions/interactions and to withdraw from participation at any time.

Before any protocol-specific procedures can be carried out, the consenting professional will fully explain the aspects of patient privacy concerning research specific information. In addition to signing the IRB Informed Consent, all patients must agree to the Research Authorization component of the informed consent form.

Each participant and consenting professional will sign the consent form. The participant must receive a copy of the signed informed consent form.

19.0 REFERENCES

1. Yu, H.A., et al., *Acquired Resistance of EGFR-Mutant Lung Cancer to a T790M-Specific EGFR Inhibitor: Emergence of a Third Mutation (C797S) in the EGFR Tyrosine Kinase Domain.* JAMA Oncol, 2015. **1**(7): p. 982-4.
2. Yu, H.A., et al., *Analysis of tumor specimens at the time of acquired resistance to EGFR-TKI therapy in 155 patients with EGFR-mutant lung cancers.* Clin Cancer Res, 2013. **19**(8): p. 2240-7.
3. Janne, P.A., Wang, X. F., Socinski J., Crawford, M., Capelletti, M., Edelman, M.J., Villalona-Calero, M.A., Kratzke, R.A., Vokes, E.E., Miller, V.A. *Randomized phase II trial of erlotinib (E) alone or in combination with carboplatin/paclitaxel (CP) in never or light former smokers with advanced lung adnocarcinoma: CALGB 30604.* Journal of Clinical Oncology, 2012. **39**(17):2063-9.
4. Wu, Y.L., et al., *Dacomitinib versus gefitinib as first-line treatment for patients with EGFR-mutation-positive non-small-cell lung cancer (ARCHER 1050): a randomised, open-label, phase 3 trial.* Lancet Oncol, 2017. **18**(11): p. 1454-1466.
5. Mok, T.S., et al., *Osimertinib or Platinum-Pemetrexed in EGFR T790M-Positive Lung Cancer.* N Engl J Med, 2017. **376**(7): p. 629-640.
6. Soria, J.C., et al., *Osimertinib in Untreated EGFR-Mutated Advanced Non-Small-Cell Lung Cancer.* N Engl J Med, 2018. **378**(2): p. 113-125.
7. Thress, K.S., et al., *Acquired EGFR C797S mutation mediates resistance to AZD9291 in non-small cell lung cancer harboring EGFR T790M.* Nat Med, 2015. **21**(6): p. 560-2.
8. Ercan, D., et al., *EGFR Mutations and Resistance to Irreversible Pyrimidine-Based EGFR Inhibitors.* Clin Cancer Res, 2015. **21**(17): p. 3913-23.
9. Yu, H.A., et al., *Phase 1 study of twice weekly pulse dose and daily low-dose erlotinib as initial treatment for patients with EGFR-mutant lung cancers.* Ann Oncol, 2017. **28**(2): p. 278-284.
10. Stein, S., et al., *Mathematical modeling identifies optimum lapatinib dosing schedules for the treatment of glioblastoma patients.* PLoS Comput Biol, 2018. **14**(1): p. e1005924.
11. Chakrabarti, S. and F. Michor, *Pharmacokinetics and Drug Interactions Determine Optimum Combination Strategies in Computational Models of Cancer Evolution.* Cancer Res, 2017. **77**(14): p. 3908-3921.
12. Chakrabarti, S. and F. Michor, *Pharmacokinetics and Drug Interactions Determine Optimum Combination Strategies in Computational Models of Cancer Evolution.* Cancer Research, 2017. **77**(14): p. 3908-3921.
13. Brown, K., et al., *Population pharmacokinetics and exposure-response of osimertinib in patients with non-small cell lung cancer.* British Journal of Clinical Pharmacology, 2017. **83**(6): p. 1216-1226.14. Ramalingham, SS., et al., *Osimertinib As First-Line Treatment of EGFR Mutation-Positive Advanced Non-Small-Cell Lung Cancer. J. Clin Oncol. 2018.* 36(9):841-849.

15. Yang JC, Kim DW, Kim SW, Cho BC, Lee JS, Ye X, Yin X, Yang Z, Jiang H, Ahn MJ. Osimertinib activity in patients (pts) with leptomeningeal (LM) disease from non-small cell lung cancer (NSCLC): Updated results from BLOOM, a phase I study. Journal of Clinical OOncology 2016 34:15_suppl, 9002-9002.
