# Supplementary material for: Identification of optimal dosing schedules of dacomitinib and osimertinib for a phase I/II trial in advanced EGFR-mutant non-small cell lung cancer
Source: Nat Commun. 2021 Jun 17;12:3697. doi: 10.1038/s41467-021-23912-4 (PMC8211846; doi:10.1038/s41467-021-23912-4)
Supplement: Supplementary file 3 — Reporting Summary [file 41467_2021_23912_MOESM3_ESM.pdf]

nature research

Corresponding author(s): Franziska Michor and Helena Yu

Last updated by author(s): Jan 10, 2021

Reporting Summary

Nature Research wishes to improve the reproducibility of the work that we publish. This form provides structure for consistency and transparency in reporting. For further information on Nature Research policies, see our Editorial Policies and the Editorial Policy Checklist.

Statistics

For all statistical analyses, confirm that the following items are present in the figure legend, table legend, main text, or Methods section.

n/a	Confirmed	
☐	☒	The exact sample size (*n*) for each experimental group/condition, given as a discrete number and unit of measurement
☐	☒	A statement on whether measurements were taken from distinct samples or whether the same sample was measured repeatedly
☐	☒	The statistical test(s) used AND whether they are one- or two-sided<br>*Only common tests should be described solely by name; describe more complex techniques in the Methods section.*
☐	☒	A description of all covariates tested
☐	☒	A description of any assumptions or corrections, such as tests of normality and adjustment for multiple comparisons
☐	☒	A full description of the statistical parameters including central tendency (e.g. means) or other basic estimates (e.g. regression coefficient) AND variation (e.g. standard deviation) or associated estimates of uncertainty (e.g. confidence intervals)
☐	☒	For null hypothesis testing, the test statistic (e.g. *F*, *t*, *r*) with confidence intervals, effect sizes, degrees of freedom and *P* value noted<br>*Give P values as exact values whenever suitable.*
☒	☐	For Bayesian analysis, information on the choice of priors and Markov chain Monte Carlo settings
☒	☐	For hierarchical and complex designs, identification of the appropriate level for tests and full reporting of outcomes
☒	☐	Estimates of effect sizes (e.g. Cohen's *d*, Pearson's *r*), indicating how they were calculated

Our web collection on statistics for biologists contains articles on many of the points above.

Software and code

Policy information about availability of computer code

Data collection	Data from experiments were collected using R Software 3.5 or newer versions. Drug-concentrations were simulated using Ubiquity (V1), a published R package with C+ dependencies.
Data analysis	Data was analyzed using R Software 3.5 or newer versions. All code can be found in https://github.com/Michorlab/NSCLC_OsimDacoOptimization.

For manuscripts utilizing custom algorithms or software that are central to the research but not yet described in published literature, software must be made available to editors and reviewers. We strongly encourage code deposition in a community repository (e.g. GitHub). See the Nature Research guidelines for submitting code & software for further information.

Data

Policy information about availability of data

All manuscripts must include a data availability statement. This statement should provide the following information, where applicable:

- Accession codes, unique identifiers, or web links for publicly available datasets
- A list of figures that have associated raw data
- A description of any restrictions on data availability

All data is available on GitHub at Michorlab/NSCLC_OsimDacoOptimization.

Field-specific reporting

Please select the one below that is the best fit for your research. If you are not sure, read the appropriate sections before making your selection.

- [x] Life sciences
- [ ] Behavioural & social sciences
- [ ] Ecological, evolutionary & environmental sciences

For a reference copy of the document with all sections, see nature.com/documents/nr-reporting-summary-flat.pdf

Life sciences study design

All studies must disclose on these points even when the disclosure is negative.

Sample size	No sample size calculation was performed. Since a Phase I is small by design, we fixed study size to 34 patients before recruiting.
Data exclusions	Apart from following subject exclusion criteria as detailed in the protocol, no other data was excluded from analysis.
Replication	Results cannot be reproduced in this study.
Randomization	No randomization in Phase Ib study since all patients were allocated to same arm.
Blinding	Blinding was not possible.

Reporting for specific materials, systems and methods

We require information from authors about some types of materials, experimental systems and methods used in many studies. Here, indicate whether each material, system or method listed is relevant to your study. If you are not sure if a list item applies to your research, read the appropriate section before selecting a response.

Materials & experimental systems

n/a	Involved in the study	
[ ]	[x]	Antibodies
[ ]	[x]	Eukaryotic cell lines
[x]	[ ]	Palaeontology and archaeology
[x]	[ ]	Animals and other organisms
[ ]	[x]	Human research participants
[ ]	[x]	Clinical data
[x]	[ ]	Dual use research of concern

Methods

n/a	Involved in the study	
[x]	[ ]	ChIP-seq
[x]	[ ]	Flow cytometry
[x]	[ ]	MRI-based neuroimaging

Antibodies

Antibodies used

Antibodies against EGFR (4267), p-EGFR Y1068 (3777), extracellular signal-regulated kinase (ERK) (9102), phosphorylated-ERK (p-ERK) T202/Y204 (9101), Akt (4691), pAktS473 (4060), S6 ribosomal protein (S6) (2217), phosphor-S6 ribosomal protein S235/236 (pS6) (4858), cleaved PARP (9541) and glyceraldehyde-3-phosphate dehydrogenase (GAPDH) (2118) were purchased from Cell Signaling Technology.

Validation

Please see below (Table S3):
Antibody (Product No) and publication of antibody validation
EGFR (4267) PMID: 12063263
p-EGFR Y1068 (3777) PMID: 12063263
ERK (9102) PMID: 9038193
p-ERK T202/Y204 (9101) PMID: 8622663
Akt (4691) PMID: 10864894
pAktS473 (4060) PMID: 10864894
S6 ribosomal protein (2217) PMID: 15960972
pS6 (4858) PMID: 19029981
cleaved PARP (9541) PMID: 11154281
GAPDH (2118) PMID: 17488287

Eukaryotic cell lines

Policy information about cell lines

Cell line source(s)	PC9 cells were purchased from RIKEN Cell Bank (Tsukuba, Ibaraki Prefecture, Japan) and were cultured in Gibco(R) RPMI (Life Technologies(R), Carlsbad, CA, USA) medium with 10% heat-inactivated FBS (Sigma(R), St. Louis, MO, USA). PC9-DRH, harboring both the single-mutant (Del) and double-mutant (Del/T790M) alleles, is a pool of cells derived from PC9 parental line that was selected after treatment with gradually increasing concentrations of dacomitinib up to 2 uM. PC9-DRH EGFR alleles consist of 70% Del/T790M and 30% Del. PC9-DRH cells were cultured in Gibco(R) RPMI medium with 10% FBS, and maintained in dacomitinib (2 uM) (Planken et al JMC). PC9R NRAS, harboring 40% NRAS Q61K mutation and sensitive to treatment with the combination of a third gen EGFR TKI (e.g. osimertinib) plus a MEK inhibitor (e.g. selumetinib), is a pool of cells derived from PC9 parental line that was selected after treatment with gradually increasing concentrations of a third generation EGFR TKI PF-06747775 up to 1 uM. PC9R NRAS cells were cultured in Gibco(R) RPMI medium with 10% FBS, and maintained in PF-06747775 (1 uM). PC9R C797S cells...
Authentication	Cell lines were authenticated by CellCheck 16 assay by IDEXX Bioanalytics, which uses 16 STR markers
Mycoplasma contamination	Cell lines were confirmed as mycoplasma negative by Stat-Myco assay by IDEXX Bioanalytics, which uses real-time PCR to detect mycoplasma contamination.
Commonly misidentified lines (See ICLAC register)	No commonly misidentified cell lines were used in the study.

Human research participants

Policy information about studies involving human research participants

Population characteristics	All patients must have: Written informed consent, Advanced biopsy-proven metastatic non-small cell lung cancer, Somatic activating mutation in EGFR in pre-treatment biopsy, No prior standard or experimental EGFR inhibitor treatment (osimertinib, dacomitinib, gefitinib, afatinib, erlotinib), Archival tissue available from a tumor biopsy or willing to undergo a tumor biopsy prior to study initiation. Measurable (RECIST 1.1) indicator lesion not previously irradiated, Karnofsky performance status (KPS) ≥ 70%, Age >18 years old, Adequate organ function, Ability to swallow oral medications
Recruitment	Subjects included patients treated at Memorial Sloan Kettering appropriate for first-line EGFR-directed targeted therapy. There was no external recruitment. Patients were identified by their primary oncologists as potentially eligible for the study. Verbal and written informed consent was obtained.
Ethics oversight	Study approved by the institutional review board at Memorial Sloan Kettering Cancer Center.

Note that full information on the approval of the study protocol must also be provided in the manuscript.

Clinical data

Policy information about clinical studies

All manuscripts should comply with the ICMJEguidelines for publication of clinical research and a completedCONSORT checklist must be included with all submissions.

Clinical trial registration	NCT03810807
Study protocol	Clinical Protocol included with submission
Data collection	Patients were enrolled between January 2019 and May 2020 and database lock was 6/19/2020. Data collection and storage was all electronic
Outcomes	Primary endpoint of the study was identification of the recommended phase 2 dose of the combination of osimertinib and dacomitinib. Dose Limiting Toxicities are adverse events (graded according to CTCAE v5) experienced in the first cycle of treatment (i.e. 4 weeks).<br><br>Secondary: Best overall response rate (confirmed partial and complete responses) will be assessed as part of this study. All responses must be confirmed on subsequent scan to be considered a confirmed response.Tumor response will be assessed using RECIST 1.1. The same method of assessment (CT scan or MRI) and the same technique (i.e. with or without contrast) should try to be used to characterize each identified and reported lesion at baseline, on study and at end of treatment.
